# Dual stimulus-responsive renewable nanoadsorbent for selective adsorption of low-density lipoprotein in serum

**DOI:** 10.1093/rb/rbae045

**Published:** 2024-04-29

**Authors:** Chen Guo, Xinbang Jiang, Xiaofang Guo, Zhuang Liu, Biao Wang, Yunzheng Du, Ziying Tian, Zimeng Wang, Lailiang Ou

**Affiliations:** Key Laboratory of Bioactive Materials, Ministry of Education, College of Life Science, Nankai University, Tianjin 300071, China; Key Laboratory of Bioactive Materials, Ministry of Education, College of Life Science, Nankai University, Tianjin 300071, China; Key Laboratory of Bioactive Materials, Ministry of Education, College of Life Science, Nankai University, Tianjin 300071, China; Key Laboratory of Bioactive Materials, Ministry of Education, College of Life Science, Nankai University, Tianjin 300071, China; Key Laboratory of Bioactive Materials, Ministry of Education, College of Life Science, Nankai University, Tianjin 300071, China; Key Laboratory of Bioactive Materials, Ministry of Education, College of Life Science, Nankai University, Tianjin 300071, China; Key Laboratory of Bioactive Materials, Ministry of Education, College of Life Science, Nankai University, Tianjin 300071, China; Key Laboratory of Bioactive Materials, Ministry of Education, College of Life Science, Nankai University, Tianjin 300071, China; Key Laboratory of Bioactive Materials, Ministry of Education, College of Life Science, Nankai University, Tianjin 300071, China

**Keywords:** hyperlipemia, blood lipids, bionic nanoadsorbent, photoregenerative, micromagnetic microfluidics

## Abstract

Selective removal of ultra-high low-density lipoprotein (LDL) from the blood of hyperlipemia patients using hemoperfusion is considered an efficient method to prevent the deterioration of atherosclerotic cardiovascular disease. Based on the exceptional structure–function properties of multistimulus-responsive materials, we developed a magnetic photorenewable nanoadsorbent (Fe_3_O_4_@SiO_2_@Azo-COOH) with outstanding selectivity and regenerative characteristics, featuring functionalized azobenzene as the ligand. The dual-stimulus response endowed Fe_3_O_4_@SiO_2_@Azo-COOH with rapid separation and photoregenerative properties. The adsorbent demonstrated excellent removal efficiency of LDL with an adsorption capacity of 15.06 mg/g, and highly repetitive adsorption performance (≥5 cycles) under irradiation. Fe_3_O_4_@SiO_2_@Azo-COOH also exhibited remarkable adsorption properties and selectivity in human serum, with adsorption capacities of 10.93, 21.26 and 9.80 mg/g for LDL, total cholesterol and triglycerides and only 0.77 mg/g for high-density lipoprotein (HDL), resulting in a 93% selective adsorption difference (LDL/HDL). Complete green regeneration of the nanoadsorbent was achieved through a simple regeneration process, maintaining a recovery rate of 99.4% after five regeneration experiments. By combining dynamic perfusion experiment with micromagnetic microfluidics, the LDL content decreased by 16.6%. Due to its superior adsorption capacity and regenerative properties, the dual stimulus-responsive nanosorbent is considered a potential hemoperfusion adsorbent.

## Graphical Abstract

**Figure:**
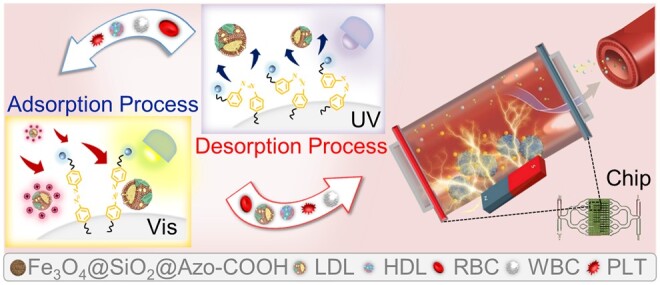

## Introduction 

For decades, atherosclerotic cardiovascular diseases caused by hyperlipemia (HLP) have garnered increasing attention [1, 2]. An important clinical indicator for determining HLP is the abnormal increase in LDL levels [3–5]. As a crucial serum lipoprotein, LDL plays an essential role in the transport and metabolism of cholesterol [6]. Dysregulation of LDL catabolism leads to the accumulation of LDL particles in the inner layer of the arterial wall, forming fatty acid deposits and atherosclerotic plaques, ultimately resulting in coronary artery disease [7]. Although dietary control and lipid-lowering medications (e.g. statins and niacin) [8, 9] have made significant strides in treating of patients with HLP, the dual challenges of pronounced side effects (e.g. acute coronary syndrome [10], muscle weakness [11], muscle pain [12] and low efficacy) from existing treatments and inefficacy in managing patients with LDL levels above 300 mg/dl [13] pose significant obstacles to HLP treatment. Studies have indicated that selectively lowering LDL level while maintaining HDL with anti-atherosclerotic properties in patients represents a promising clinical treatment strategy for HLP [14, 15]. Encouragingly, hemopurification-based adsorption has shown to be an effective therapy for treating HLP, demonstrating not only good adsorption efficiency for pathogenic LDL and selectivity adsorption resisting HDL, but also a reduction in toxicities, particularly effective for patients with familial hypercholesterolemia [16] and those who fail to achieve complete recovery through diet and drug therapy.

Obviously, the removal efficiency of hemoperfusion depends on the adsorption properties of the LDL adsorbent [17]. Typically, the adsorbent consists of matrix and functional ligands (or additional arms) [18]. As the section directly contacting the adsorbate, the ligand is directly related to the removal efficiency. In current studies, LDL clearance has been performed through antibody–antigen specific interactions [19, 20], electrostatic binding [21] and hydrophobic actions [22], with common ligands including anti-human phospholipids [23], *β*-heparin [16], lipoprotein antibodies [24], chondroitin sulphate [25], hyaluronic acid [26] and cyclodextrins [27]. However, there are still some unsatisfactory problems with current methods, such as poor adsorption efficiency and selectivity due to the low graft density of the ligand and poor reusability caused by the nature of the material itself, thus improving the treatment or developing new adsorbent materials would be valuable. LDL micro nanoparticles (*d* = 20–25 nm) [28] consist of a nonpolar lipid core containing triglycerides (TG) and cholesterol, surrounded by a shell composed of a phospholipid monolayer, unesterified cholesterol and apolipoprotein [29]. Numerous studies have demonstrated that LDL tends to adhere to hydrophobic surface or adsorbent and combines more preferentially with negatively charged groups (carboxyl, sulphonate, phosphate, etc.) [30, 31]. Therefore, novel negatively charged hydrophobic adsorbents are considered ideal for the removal of LDL.

Stimulus-responsive polymer (SRP) materials undergo changes in physical form and chemical properties in response to small alterations in the external environment (e.g. temperature, pH, light or magnetic field) [32]. When immobilized, SRP materials manufactured in various forms and compositions, offer new opportunities for separating products generated by the biotechnology industry. Among the numerous stimuli responsiveness, magnetic and photo stimulation have garnered significant attention due to their cleanliness and ability for remote control [33–35], particularly in applications within separation science. Of particular interest, azobenzene is a common photosensitive group exhibiting photoisomerization behavior that can reverse surface wettability, which is highly advantageous for preparingrenewable adsorbents for blood lipids [36, 37]. Therefore, azobenzene is regarded as an excellent functional ligand for constructing renewable LDL blood perfusion adsorbents.

In this study, we prepared a dual stimulus-responsive regenerable nanoadsorbent for selectively clearing pathogenic blood lipids through intensive investigation of regenerative properties. The synthesis process firstly involved preparing azobenzene functional monomers and functionalized magnetic carriers. Then, the N-hydroxysuccinimide (NHS) terminated azobenzene functional monomers were grafted onto the surface of the amino-modified magnetic carriers through amino-NHS esterification reaction, enabling the nanoadsorbent with dual photomagnetic response. Finally, to enhance adsorption selectivity between LDL and HDL, the nanoparticles were modified via thiol-alkene click reaction to obtain a negative-charged hemoperfusion adsorbent. The adsorbent not only possessed good adsorption properties for blood lipids but also exhibited good selectivity for anti-atherosclerotic HDL. Notably, the prepared dual stimulus-responsive adsorbent enabled rapid separation utilizing magnetic responsiveness and flexible recycling through photo-responsiveness, demonstrating good regenerative properties. Combined with micro-magnetic microfluidic technology for dynamic hemoperfusion experiments, serum lipid content was significantly reduced, indicating the potential of the material for hemoperfusion. To the best of our knowledge, a nanoadsorbent with dual photomagnetic response for clearing LDL has not been reported, making this bionic nanoadsorbent a novel application in the field of blood perfusion.

## Experiment

### Materials and reagents

The materials and reagents utilized in this work, along with their associated purification and synthesis methods, were described in the Supplementary Information.

### Synthesis of Fe_3_O_4_@SiO_2_-NH_2_ nanoparticles with photoregenerative azobenzene ligand via amination reaction (briefly Fe_3_O_4_@SiO_2_@Azo)

Due to the high reactivity between amino and NHS, the photoregenerative Fe_3_O_4_@SiO_2_@Azo nanoparticles were prepared by “active” Fe_3_O_4_@SiO_2_-NH_2_ and AL_10_-OSU [38]. Briefly, Fe_3_O_4_@SiO_2_-NH_2_ (0.3 g) was dispersed ultrasonically in tetrahydrofuran (THF, 180 ml) in a three-neck round-bottom flask, followed by the addition of AL_10_-OSU (540 mg, 0.98 mmol) and ultrasonic for 5 min. Triethylamine was then added dropwise, and the reaction proceeded at room temperature for 24 h. Finally, the nanoparticles were magnetically separated, washed (THF and ethanol) and dried at 40°C under vacuum for 48 h to obtain a brownish-black powder (Fe_3_O_4_@SiO_2_@Azo), showing 7.1% weight increase compared to Fe_3_O_4_@SiO_2_-NH_2_ nanoparticles.

### Synthesis of highly selective photoresponsive nanoparticles (Fe_3_O_4_@SiO_2_@Azo-COOH) via click reaction

Since “click” reaction was found to proceed with high efficiency in a short time and produced almost no by-products, the negative azobenzene modified nanoparticles were constructed by combining Fe_3_O_4_@SiO_2_@Azo with carboxyl groups through the “click” reaction of alkene with thiol [39]. The synthesis process involved dispersing Fe_3_O_4_@SiO_2_@Azo ultrasonically in THF, bubbling argon into the mixture for 10 min at 25°C, adding 3-mercaptopropionic acid and bubbling argon for another 10 min. Triethylamine was then added dropwise with further argon aeration, and the reaction solution was stirred at 25°C for 24 h. The resulting nanoparticles were magnetically separated, washed (methanol) until the supernatant became clear, and then, dried under vacuum at 40°C for 48 h to obtain carboxylated magnetic photoresponsive nanoparticles (Fe_3_O_4_@SiO_2_@Azo-COOH), showing a 4.9% weight increase compared to Fe_3_O_4_@SiO_2_@Azo.

### Characterization

The sample was characterized using a magnetic analysis system, fourier-transform infrared spectroscopy (FTIR), scanning electron microscope (SEM) and transmission electron microscope (TEM). The hydrophobicity of the nanoparticles' surface was assessed through the water contact angle (WCA, evaluation via thin films made of the prepared nanoparticles) and an aqueous dispersion stability test. Blood lipid levels were tested using a blood biochemical analyzer.

UV–Vis spectrometer was employed to evaluate the UV–Vis spectra of the nanoadsorbent. The photochemical isomerization of the samples was achieved by irradiation with a UV lamp (365 nm, 6 W) at 25°C until reaching the photostationary condition, followed by irradiation with a visible light lamp with a filter (*λ* > 510 nm, 16 W).

### Adsorption performance

The static adsorption of LDL *in vitro* was tested using a LDL solution (concentration > 4.2 mmol/l) in phosphate-buffered saline (PBS) and in the human serum of HLP patients to evaluate the clearance performance of the magnetic photorenewable nanoparticles. The adsorption procedure involved dispersing the nanoadsorbent ultrasonically in 0.25 ml PBS in a series of 2 ml Eppendorf tubes, followed by the addition of 0.25 ml LDL solution (or serum). The tubes were then incubated for 2 h at 37°C under 160 rpm. After the reaction, the solution was collected through magnetic separation, and the levels of HDL, LDL, TG and total cholesterol (TCH) were determined using a blood biochemical analyzer.

*In vitro* dynamic LDL adsorption testing was conducted in a hemoperfusion chip inspired by the microarchitecture of the spleen. According to the literature [40], the modified chip was prepared from poly(methylmethacrylate) (PMMA) material with a three-layer channel structure. HLP patients' serum and magnetic photoregenerative nanoparticles were injected through the lower chip channels, and mixed thoroughly in the chip channels for lipid removal, followed by separation and collection of the nanoparticles via the magnetic effect from the middle and upper chip channels. The amount of nanoadsorbent used in the dynamic adsorption of hemoperfusion chip was 1.5 times greater than in static adsorption. Similarly, the levels of blood lipids were determined by a blood biochemistry analyzer.

The clearance efficiency of Fe_3_O_4_@SiO_2_@Azo-COOH was calculated using Equation (1):

(1) $$Q_{c}=(C_{0}-C_{1})\times {\;K}_{\mathrm{TF}\;}\times 10\times V_{t}/m$$

where *Q* represents the adsorption performance of the prepared nanoadsorbent (mg/g). *C*_0_ denotes the blood lipid levels of the primary solution (mmol/L). *C*_1_ represents the blood lipid levels of the final solution (mmol/l). *V_t_* is the total volume of solution (l). *m* is the quantity of adsorbent (g). *K*_***TF***_ denotes the transformation factor of each blood lipids. LDL, HDL or TCH *K*_***TF***_ is ∼38.67; TG *K*_***TF***_ is around 88.57.

### Photocontrolled adsorption processes and binding relationship studies

According to previous literature [37], the photocontrolled adsorption–desorption process of Fe_3_O_4_@SiO_2_@Azo-COOH was carried out through adsorption experiments with cyclic light conversion under UV light irradiation. A series of 2 ml Eppendorf tubes containing adsorption–desorption samples (Fe_3_O_4_@SiO_2_@Azo-COOH, 0.005 g) were added to LDL solution (0.5 ml). The mixture was initially incubated for adsorption in the dark at 37°C for 120 min under 160 rpm. After the 2-hour incubation, the first group of samples, namely A_1_-Fe_3_O_4_@SiO_2_@Azo-COOH, was removed, and the adsorption solution was collected by magnetic separation for measuring the LDL level. The remaining samples were then irradiated with UV light for incubation desorption. Following another two-hour irradiation, the second group of samples namely D_1_-Fe_3_O_4_@SiO_2_@Azo-COOH was removed, and the adsorption solution was collected for measuring the LDL level. Subsequently, the remaining samples were incubated under continuous photo-switching cyclic irradiation of UV-dark incubation through the above process, sequentially named An-Fe_3_O_4_@SiO_2_@Azo-COOH and D_*n*_-Fe_3_O_4_@SiO_2_@Azo-COOH (*n* = 2, 3, 4, 5), with the relative adsorption–desorption states listed in Table 1. The LDL adsorption capacity of the samples was determined based on the concentration changes of the supernatant. Meanwhile, the photocontrolled adsorption–desorption performance of Fe_3_O_4_@SiO_2_@Azo-COOH was monitored by TEM and FTIR analysis. The photo-induced adsorption–desorption property of Fe_3_O_4_@SiO_2_@Azo-COOH was also investigated in the serum of patients with HLP. The adsorption–desorption procedures for the various samples were followed as described above, and the changes in the concentrations of LDL, TG, TCH and HDL in the different states were determined using a blood biochemistry analyzer.

**Table 1.** Photocontrolled adsorption–desorption procedure of Fe_3_O_4_@SiO_2_@Azo-COOH in LDL solution

Sample	Photo-switching cycles										State
	Off	On	Off	On	Off	On	Off	On	Off	On	
A_1_-Fe_3_O_4_@SiO_2_@Azo-COOH	**+**	**−**	**−**	**−**	**−**	**−**	**−**	**−**	**−**	**−**	**
D_1_-Fe_3_O_4_@SiO_2_@Azo-COOH	**+**	**+**	**−**	**−**	**−**	**−**	**−**	**−**	**−**	**−**	*
A_2_-Fe_3_O_4_@SiO_2_@Azo-COOH	**+**	**+**	**+**	**−**	**−**	**−**	**−**	**−**	**−**	**−**	**
D_2_-Fe_3_O_4_@SiO_2_@Azo-COOH	**+**	**+**	**+**	**+**	**−**	**−**	**−**	**−**	**−**	**−**	*
A_3_-Fe_3_O_4_@SiO_2_@Azo-COOH	**+**	**+**	**+**	**+**	**+**	**−**	**−**	**−**	**−**	**−**	**
D_3_-Fe_3_O_4_@SiO_2_@Azo-COOH	**+**	**+**	**+**	**+**	**+**	**+**	**−**	**−**	**−**	**−**	*
A_4_-Fe_3_O_4_@SiO_2_@Azo-COOH	**+**	**+**	**+**	**+**	**+**	**+**	**+**	**−**	**−**	**−**	**
D_4_-Fe_3_O_4_@SiO_2_@Azo-COOH	**+**	**+**	**+**	**+**	**+**	**+**	**+**	**+**	**−**	**−**	*
A_5_-Fe_3_O_4_@SiO_2_@Azo-COOH	**+**	**+**	**+**	**+**	**+**	**+**	**+**	**+**	**+**	**−**	**
D_5_-Fe_3_O_4_@SiO_2_@Azo-COOH	**+**	**+**	**+**	**+**	**+**	**+**	**+**	**+**	**+**	**+**	*

***+*** The sample was on the photo-switching cycle.

**−** The sample was not on the photo-switching cycle.

** The sample was in an adsorption-state.

* The sample was in a desorption-state.

To investigate the binding relationship of the prepared adsorbent with the adsorbates (HDL, LDL, TG and TCH) in serum, the potential interaction adsorption mechanisms between them were explored through variations in the adsorption rate and examination of the thermodynamic properties of the adsorption process. For kinetic experiments, the adsorption rate of the adsorbent was explored by varying the adsorption time. Fe_3_O_4_@SiO_2_@Azo-COOH (0.005 g) was mixed with HLP serum (0.5 ml), and incubated at 37°C under 160 rpm for different periods of time. For isotherm experiments, the thermodynamic properties of the adsorbent were investigated by altering the initial adsorption concentration. Fe_3_O_4_@SiO_2_@Azo-COOH (0.005 g) was added to different initial concentrations of HLP serum (0.5 ml), and incubated at 37°C under 160 rpm for 2 h. After adsorption, the lipid levels were determined using a blood biochemistry analyzer, and the adsorption capacity was measured according to Equation (1).

### Recycling and reusability of the adsorbent

The recycling and reusability of the photorenewable adsorbent were determined through a continuous adsorption–desorption process, involving UV irradiation and elution reagent treatment. First, based on the photocontrolled adsorption–desorption studies, the effect of elution reagent treatment on the regeneration of the sample was investigated. After adsorption, the sample was processed using or without UV irradiation, and then, washed with elution reagent (2.5 M NaCl) for 3 times. Subsequently, the regeneration testing, combining photo-responsive adsorption and elution regeneration, was performed for five cycles. The recovery rate was quantified by comparing the adsorption capacity of the regenerated Fe_3_O_4_@SiO_2_@Azo-COOH for blood lipids with the original adsorption capacity.

### Biosafety assessment of the adsorbent

To verify the biosafety and assess the suitability of the prepared adsorbent for clinical application, biocompatibility was investigated through hemocompatibility analysis (blood routine testing, anticoagulant activity analysis, hemolysis testing) and cytotoxicity testing.

## Results and discussion

### Preparation and characterization of highly selective photoresponsive Fe_3_O_4_@SiO_2_@Azo-COOH nanoparticles

From a molecular structural perspective, LDL particles consist of a nonpolar lipid core rich in free cholesterol and cholesteryl esters, surrounded by a polar shell of phospholipids and apolipoprotein B-100 (apoB-100). Ligands, as crucial components of the adsorbent, play a pivotal role in the binding process with adsorbates. For the removal of electrically charged hydrophobic lipoprotein, hydrophobic and electrostatic interactions are decisive. Therefore, with an understanding of the LDL structure, azobenzene functional monomers, whose hydrophilicity changes under light stimulation (confirmed by structural characterization by ^1^H NMR), were designed at the molecular level to enable the photo-intelligent clearance of LDL. Additionally, to achieve the maximum grafting density of azobenzene ligands, azobenzene was introduced onto the “active” Fe_3_O_4_@SiO_2_-NH_2_ surface using the highly efficient amino-NHS esterification reaction. Subsequently, the nanoparticles were carboxylate-modified using the thiol-alkene click reaction to improve adsorption capacity and selectivity for HDL. Compared to the previous products, some weight changes were observed in the nanoparticles at each step (Supplementary Table S1), indicating successful modification.

**Figure 1.**
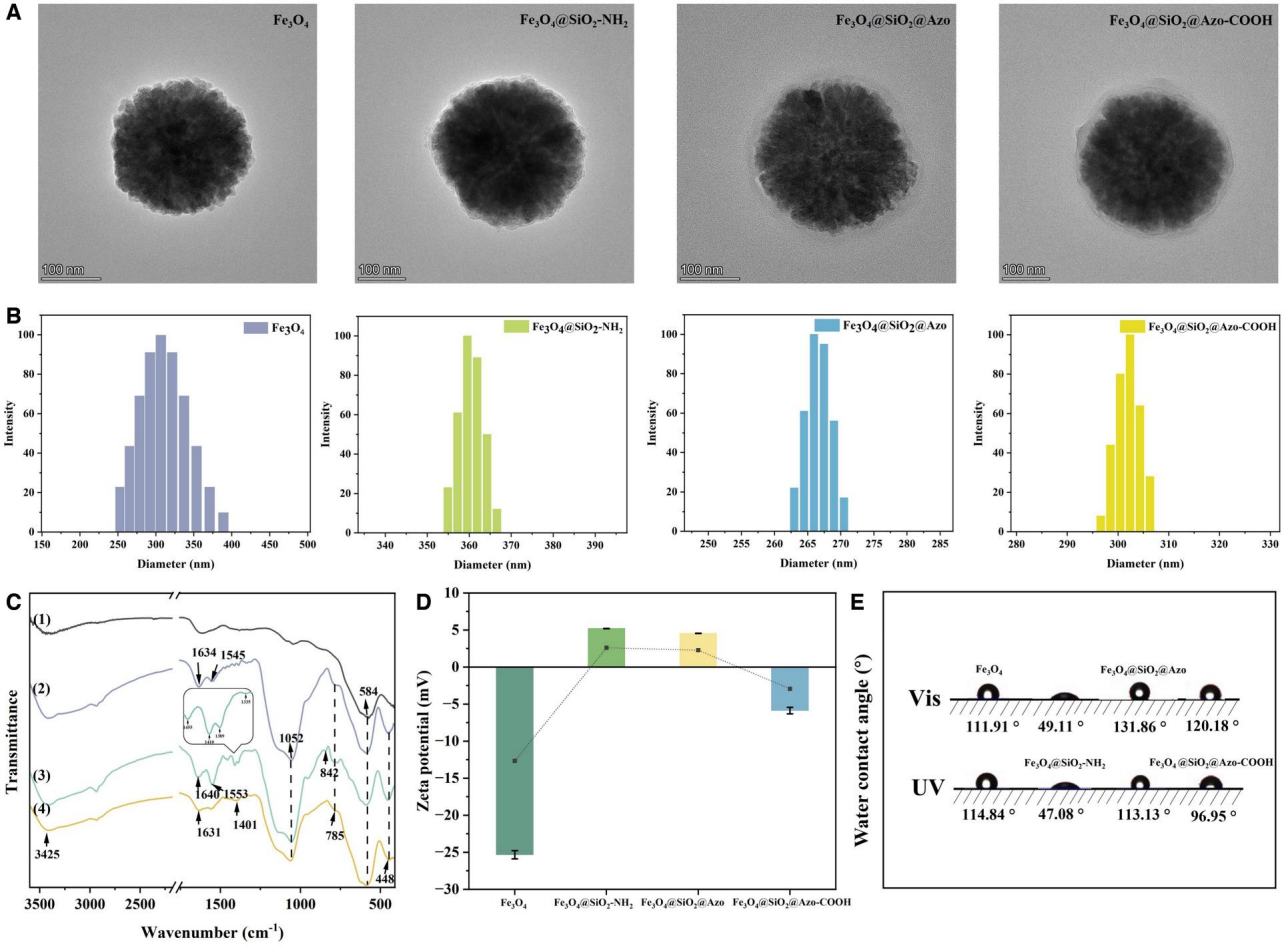
Physicochemical characterization of the samples at different stages of the synthetic procedure. (**A**) TEM images, (**B**) hydrated particle size, (**C**) FTIR spectrometer, (**D**) zeta potential test and (**E**) WCA under different light conditions of Fe_3_O_4_, Fe_3_O_4_@SiO_2_-NH_2_, Fe_3_O_4_@SiO_2_@Azo and Fe_3_O_4_@SiO_2_@Azo-COOH (*n* = 3).

The structure and surface morphology of the obtained samples were characterized via SEM, TEM and hydrodynamic diameter measurements. All nanoparticles exhibited relatively homogeneous spherical nanostructures, with diameters ranging from 225.2 to 301.3 nm (Supplementary Figure S2 and Table S1), and size distribution indices (*U*) around 1.057–1.124 (entries 1–4, Supplementary Table S1). The introduction of aminated shell layer and grafting of azobenzene led to changes in diameter, confirming the successful modification of Fe_3_O_4_. Further evidence of surface modification was demonstrated through different surface roughness observed in the magnified TEM images (Figure 1A) of unmodified and modified Fe_3_O_4_. As depicted in Figure 1B, pristine Fe_3_O_4_ nanoparticles had a hydrodynamic diameter of ∼301.9 nm, increasing to 360.4 nm after coating with an aminated-silica layer. The hydrodynamic diameters for Fe_3_O_4_@SiO_2_@Azo and Fe_3_O_4_@SiO_2_@Azo-COOH were 266.6 nm and 306.7 nm, respectively. Compared with Fe_3_O_4_@SiO_2_-NH_2_, a significant size decrease in Fe_3_O_4_@SiO_2_@Azo was observed, which was attributed to enhanced surface hydrophobicity by the grafted azobenzene ligand. Further modification with 3-mercaptopropionic acid resulted in a distinct size increase in hydrodynamic diameter due to the hydration effect on carboxyl.

In Figure 1C, the FTIR spectra depict initial Fe_3_O_4_ and its surface-modified forms, namely Fe_3_O_4_@SiO_2_-NH_2_, Fe_3_O_4_@SiO_2_@Azo and Fe_3_O_4_@SiO_2_@Azo-COOH. The Fe-O stretching peak at 576 cm^−1^ was distinctly observed for Fe_3_O_4_. Successful coating of the amino-SiO_2_-shell was evidenced by the characteristic peaks of Si-O-Si stretching (around 1052 cm^−1^) and Si-O vibration (around 785 and 448 cm^−1^) in the Fe_3_O_4_@SiO_2_-NH_2_ matrix. Additionally, the peaks around 1640 cm^−1^ and 1553 cm^−1^ emerged from the -NH_2_ stretching band and in-plane bending on the surface of the silicon sphere. The successful modification of azobenzene ligand was proved by the emergence of ring-carbon skeleton vibration peaks of benzene around 1455, 1410, 1389, 1335 cm^−1^ and 1,4-substituted out-of-plane bending vibrations of the benzene ring near 842 cm^−1^. Furthermore, successful chemical grafting was demonstrated by several characteristic absorption peaks of the amide groups around 1640 cm^−1^ (C = O stretching, amide I) and 1553 cm^−1^ (C-N-H stretching, amide II). Additionally, the carboxyl modification of Fe_3_O_4_@SiO_2_@Azo-COOH was confirmed by the carboxyl characteristic peaks of 3-mercaptopropionic acid at ∼3425 cm^−1^ (COO–H stretching), 1631 cm^−1^ (C = O stretching) and 1401 cm^−1^ (C-S-C bending vibration).

To further validate the prepared nanoparticles, different samples in our study were measured for surface potential (Figure 1D), WCA (Figure 1E) and aqueous dispersion stability (Supplementary Figure S4). The modification of azobenzene ligand onto nanoparticales caused a significant enhancement in the hydrophobicit of Fe_3_O_4_@SiO_2_@Azo, compared to Fe_3_O_4_@SiO_2_-NH_2_ (with a hydrophilic silicon layer). Subsequently, the modification of the carboxyl group led to an improvement in the electronegativity of Fe_3_O_4_@SiO_2_@Azo-COOH (Figure 1D). In Supplementary Figure S5, TGA was employed to analyze the thermal stability and weight loss of the nanoparticles. The final weight loss of Fe_3_O_4_@SiO_2_@Azo-COOH (4.2% decrease) and Fe_3_O_4_@SiO_2_@Azo (3.3% decrease) were much lower than that of Fe_3_O_4_@SiO_2_-NH_2_, suggesting increased thermo-stability of Fe_3_O_4_@SiO_2_@Azo-COOH caused by the grafting of azobenzene and carboxylate groups. Notably, the thermo-stability of Fe_3_O_4_@SiO_2_@Azo-COOH was slightly lower than that of Fe_3_O_4_@SiO_2_@Azo owing to the enhancement of the structural stability by the thiol-alkene click reaction. Additionally, the superparamagnetic performance of Fe_3_O_4_@SiO_2_@Azo-COOH was ∼70 emu/g (Supplementary Figure S6), enabling the magnetic separation of the nanoparticles from the solution, which could be completed within 29 s.

### Photoresponsivity of the nanoadsorbent

**Figure 2.**
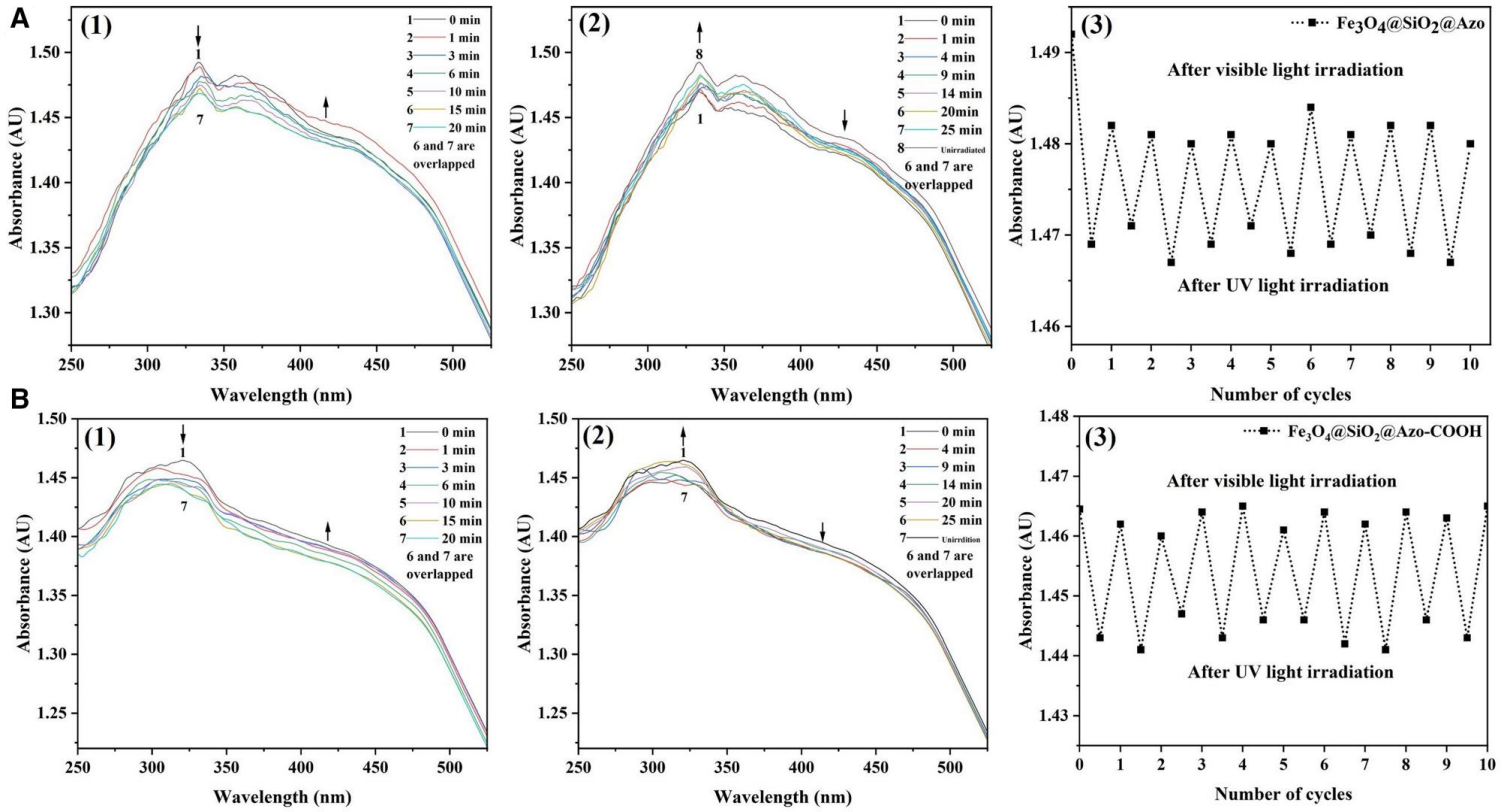
Photoresponsivity testing of samples. UV–Vis spectral changes in dependence of (**A**) Fe_3_O_4_@SiO_2_@Azo and (**B**) Fe_3_O_4_@SiO_2_@Azo-COOH at 25°C upon irradiation with 365 nm UV light (1) and upon irradiating the nanospheres at the photostationary state with visible light (*λ* > 510 nm) (2); (3) UV and visible light-induced photoisomerization cycles of the nanospheres at 25°C, in each cycle, the nanospheres were first irradiated with UV light for 20 min and then with visible light for 25 min.

The introduction of the azobenzene group to Fe_3_O_4_@SiO_2_@Azo and Fe_3_O_4_@SiO_2_@Azo-COOH endowed the samples with photoresponsive properties, resulting in altered characteristic absorption peaks after photoisomerization. The UV–Vis spectral variations of Fe_3_O_4_@SiO_2_@Azo and Fe_3_O_4_@SiO_2_@Azo-COOH were investigated, and the results were presented in Figure 2. In the spectrum of Fe_3_O_4_@SiO_2_@Azo, a distinct absorption peak at 333 nm and a relatively weak absorption peak at 417 nm [Figure 2A (1, 2)] were observed, which might be attributed to the π-π* and n-π* electronic transition of the -N = N- bond. Moreover, reversible *trans/cis* photoisomerization (cyclic process ≥10 times) was observed when Fe_3_O_4_@SiO_2_@Azo was alternately irradiated with UV and visible light [Figure 2A (3)], providing theoretical evidence for the light-controlled adsorption–desorption of the sample. Similarly, two analogous absorption peaks were identified in the spectra of negativity modified photoresponse Fe_3_O_4_@SiO_2_@Azo-COOH, albeit with different absorption sites located at 321 nm and 418 nm, respectively (Figure 2B).

Photoisomerization of azobenzene induces alterations in its *trans* and *cis* isomers, resulting in changers in dipole moment and surface wettability. These alterations in surface wettability directly contribute to the photocontrolled adsorption–desorption phenomenon for lipoprotein clearance. In addition, the presence of nano-sized forms enhances the surface roughness of the adsorbent, which can facilitate the wettability conversion of the material under external stimuli, thereby enhancing its reversible adsorption–desorption capacity. Figure 1E demonstrates the WCA of thin films prepared by dispersing nanoparticles under light irradiation. The WCA of Fe_3_O_4_@SiO_2_@Azo was 133.86°, while following UV irradiation, the WCA changed to 113.13°. Similarly, the WCA of Fe_3_O_4_@SiO_2_@Azo-COOH changed from 120.18° to 96.95°. However, the Fe_3_O_4_ and Fe_3_O_4_@SiO_2_-NH_2_ surface with ungrafted azobenzene ligands did not undergo corresponding changes.

### Adsorption experiment

The electropositivity of LDL owes to the existence of arginine and lysine clusters of apoB-100, which is at the 25% position of carboxyl terminus. Furthermore, the large amount of unesterified cholesterol and lipophilic molecules existing on apoB-100 endows LDL with considerable hydrophobicity [28]. Thus, the surface of LDL possesses both hydrophobic and electrostatic structural domains. In contrast, the major protein structural domain of HDL, apolipoprotein A-I (apoA-I), exhibits electroneutral properties owing to the equal amount of amino acid residues with different electrical properties (positively and negatively charged) [41]. Hecce, adsorbent, possessing electronegativity and hydrophobicity, was designed to combine with LDL and against HDL during the clearance procedure.

**Figure 3.**
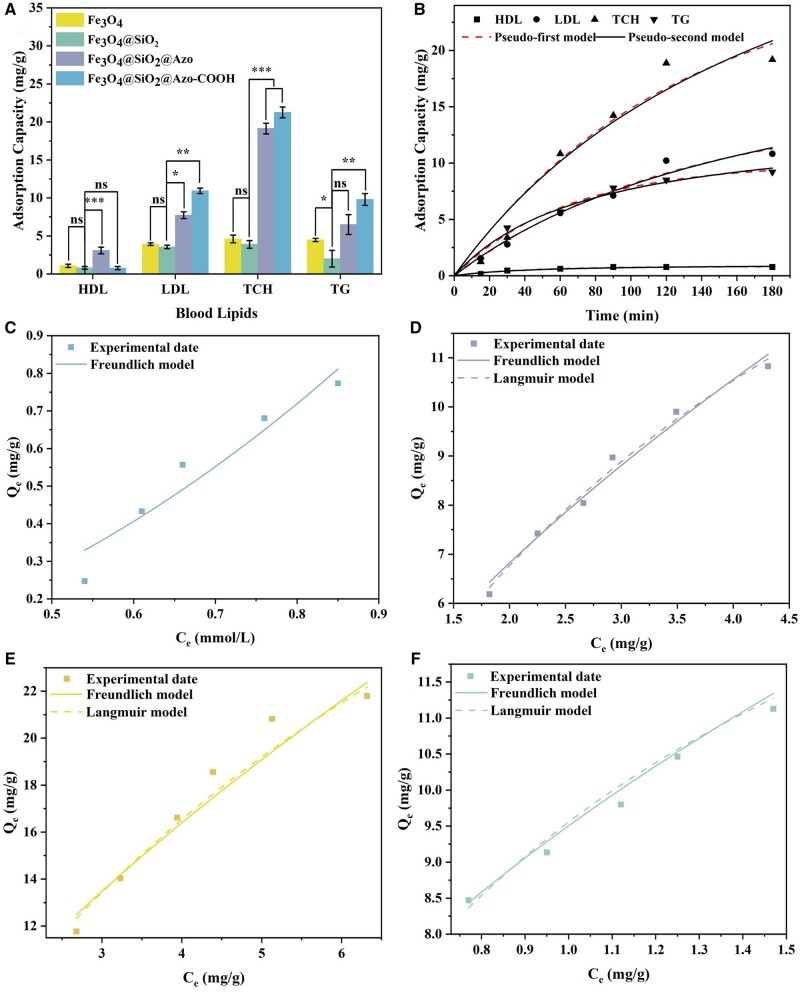
The adsorption capacity of the prepared nanoadsorbents. (**A**) Adsorption efficiency of different nanoadsorbents for blood lipids in HLP serum (*n* = 3, *<*P* < 0.05, **P < 0.01 and ***P < 0.001); (**B**) adsorption kinetics of Fe_3_O_4_@SiO_2_@Azo-COOH for blood lipids in HLP serum; adsorption isotherms of Fe_3_O_4_@SiO_2_@Azo-COOH toward (**C**) HDL, (**D**) LDL, (**E**) TCH and (**F**) TG in HLP serum (*n* = 3).

Based on the aforementioned discussion, we initially investigated the binding efficiency of the prepared adsorbent in LDL solution. The results indicated that the nanoadsorbent exhibited significant clearance properties for LDL with an adsorption capacity of 15.06 mg/g (Table 2). Furthermore, its adsorption ability and selective adsorption were studied *in vitro* through static adsorption in HLP serum (Table 2 and Figure 3A). Compared to Fe_3_O_4_@SiO_2_ (Supplementary Figure S3), the introduction of the azobenzene ligand enhanced the hydrophobic interaction between Fe_3_O_4_@SiO_2_@Azo and lipoprotein, while the electronegative modification of the adsorbent improved the selective adsorption capacity of Fe_3_O_4_@SiO_2_@Azo-COOH for HDL. The respective adsorption amounts of Fe_3_O_4_@SiO_2_ for HDL, LDL, TG and TCH was only ∼0.82, 3.56, 3.90 and 2.01 mg/g, respectively. Compared with Fe_3_O_4_@SiO_2_, the adsorption efficiency of Fe_3_O_4_@SiO_2_@Azo to HDL, LDL, TG and TCH significantly increased to 3.09, 7.73, 19.13 and 6.50 mg/g, respectively. However, owing to the poor selective adsorption effect of Fe_3_O_4_@SiO_2_@Azo, therapeutic effects for clinical HLP patients were not satisfactory. To further optimize the adsorption efficiency, electronegative modification of Fe_3_O_4_@SiO_2_@Azo based on grafted azobenzene ligands was carried out, resulting in improved adsorption efficiency for pathogenic LDL and selectivity against HDL. The clearance efficiency of Fe_3_O_4_@SiO_2_@Azo-COOH for LDL, TG and TCH was 10.93, 21.56 and 9.80 mg/g, respectively. In comparison, the binding capacity to HDL was only 0.77 mg/g, which was 75% less than Fe_3_O_4_@SiO_2_@Azo. There was a 93% difference in selective adsorption between LDL and HDL, demonstrating the successful grafting of the carboxylated azobenzene ligand. Based on its superior clearance performance and selectivity, Fe_3_O_4_@SiO_2_@Azo-COOH was regarded as a potential adsorbent for the removal of atherosclerotic factors in hemoperfusion therapy for HLP.

**Table 2.** Clearance efficiency of different nanoadsorbents in LDL solution or HLP serum

Entry	Sample	Clearance efficiency in LDL solution (mg/g)	Clearance efficiency in HLP serum (mg/g)			
			HDL	LDL	TG	TCH
1	Fe_3_O_4_	**−**	1.08 ± 0.22	3.92 ± 0.18	4.61 ± 0.50	4.49 ± 0.22
2	Fe_3_O_4_@SiO_2_	**−**	0.82 ± 0.18	3.56 ± 0.22	3.90 ± 0.50	2.01 ± 1.09
3	Fe_3_O_4_@SiO_2_@Azo	**−**	3.09 ± 0.44	7.73 ± 0.44	19.13 ± 0.71	6.50 ± 1.31
4	Fe_3_O_4_@SiO_2_@Azo-COOH	15.06 ± 0.30	0.77 ± 0.22	10.93 ± 0.36	21.56 ± 0.71	9.80 ± 0.0.79

In addition, due to the presence of a magnetic core, the adsorbent could be rapidly enriched and separated by an external magnetic field. To maximize the “bionic” functionality of the photomagnetic dual-responsive nanoadsorbent, a microfluidic chip inspired by the microstructure of the activated spleen was utilized for dynamic adsorption *in vitro* [40]. In the living spleen, as blood flow slows down and trickles from the marginal zone and red-pulp cords into the venous sinuses, the blood is filtered through the slits between neighboring endothelial cells with resident macrophages removing pathogens from the blood [42]. Thus, the microfluidic chip consisted of two adjacent connected rectangular channels, one mimicking a vascular channel (2 mm × 0.5 mm × 27 mm, width × height × length) allowing serum to pass through, and the other mimicking a venous sinus of the spleen (2 mm × 0.8 mm × 27 mm) containing intermittent or slow-flowing PBS (2 ml/min, 5 min on and 60 min off). Correspondingly, a series of open rectangular slits were also included between the two channels for connecting the blood channel and the sinus channel (2 mm × 0.34 mm × 0.34 mm, spaced every 0.43 mm). For high throughput and separation efficiency at high flow rates, the single channel into the chip was divided into four branches before the 16 magnetic separation channels (Supplementary Figure S7a).

**Figure 4.**
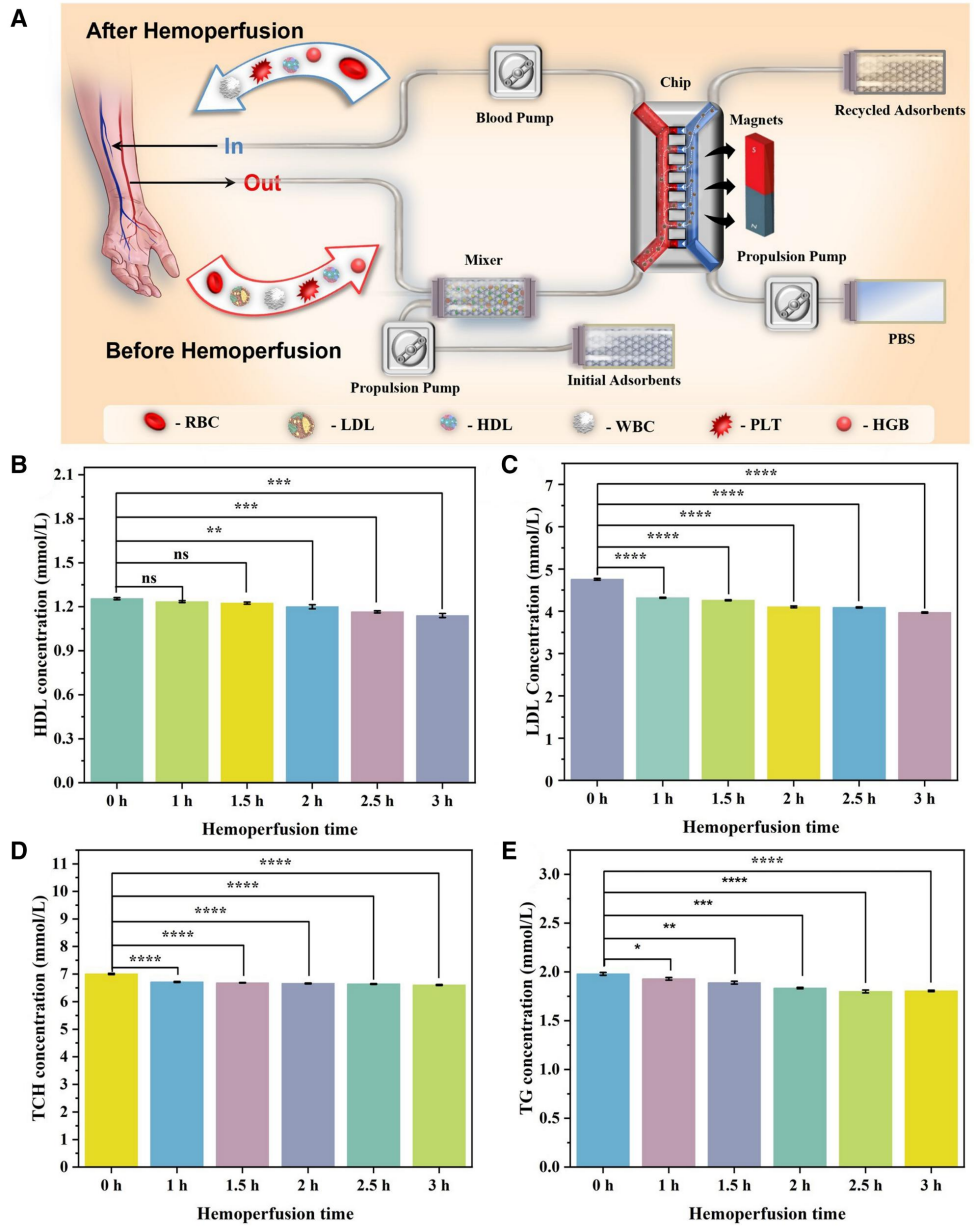
The *in vitro* evaluation for treatment efficiency of Fe_3_O_4_@SiO_2_@Azo-COOH through dynamic adsorption experiments in HLP serum. (**A**) Schematic illustration for simulated hemoperfusion process; the concentrations of (**B**) HDL, (**C**) LDL, (**D**) TCH and (**E**) TG in HLP serum after different simulated hemoperfusion times (*n* = 3, *<*P* < 0.05, **P < 0.01, ***P < 0.001 and ****P < 0.0001).

**Figure 5.**
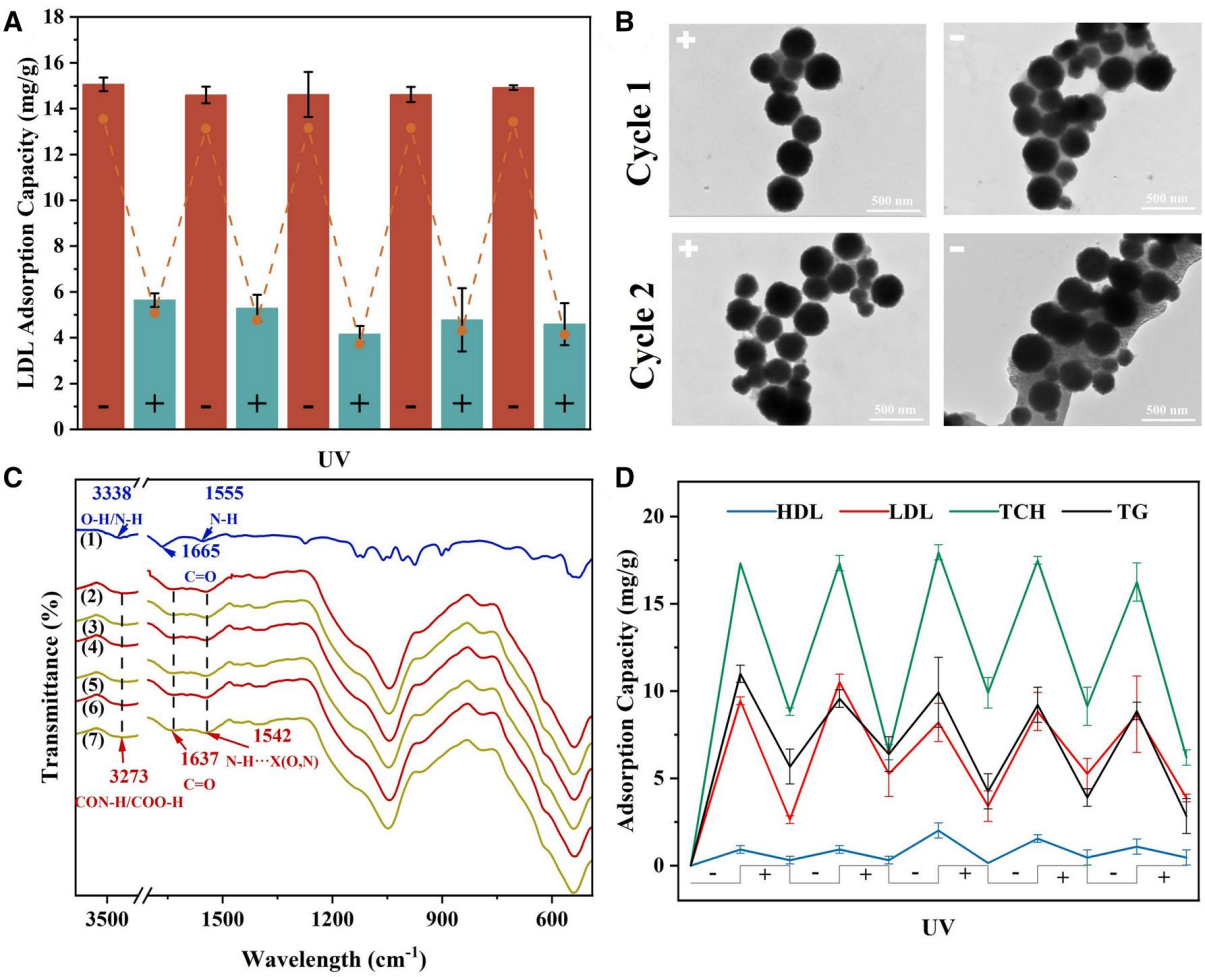
Light-controlled adsorption of the prepared nanoadsorbent. (**A**) Light-controlled adsorption efficiency of LDL on Fe_3_O_4_@SiO_2_@Azo-COOH in LDL solution (*n* = 3); (**B**) TEM images of Fe_3_O_4_@SiO_2_@Azo-COOH during light-controlled adsorption; (**C**) FTIR spectrometer of Fe_3_O_4_@SiO_2_@Azo-COOH during light-controlled adsorption [LDL(1), A_1_-Fe_3_O_4_@SiO_2_@Azo-COOH (2), D_1_-Fe_3_O_4_@SiO_2_@Azo-COOH (3), A_2_-Fe_3_O_4_@SiO_2_@Azo-COOH (4), D_2_-Fe_3_O_4_@SiO_2_@Azo-COOH (5), A_3_-Fe_3_O_4_@SiO_2_@Azo-COOH (6), D_3_-Fe_3_O_4_@SiO_2_@Azo-COOH (7)]; (**D**) light-controlled adsorption efficiency of blood lipids on Fe_3_O_4_@SiO_2_@Azo-COOH in HLP serum (*n* = 3).

To simulate the clinical blood perfusion process, a dynamic adsorption device was constructed to conduct *in vitro* dynamic adsorption experiment by utilizing the intrinsic properties of the nanoadsorbent and the special structure of the microfluidic chip (Figure 4A). In order to simulate the clinical situation, perfusion experiments were performed using serum from HLP patients (Supplementary Figure S7B and C). After 3 h of hemoperfusion perfusion, the initial concentrations of pathogenic LDL (Figure 4C), TCH (Figure 5D) and TG (Figure 4E) were significantly reduced from 4.76, 7.01 and 1.98 mmol/L to 3.97, 6.61 and 1.81 mmol/L, respectively. In contrast, HDL concentration (Figure 4B) decreased from 1.26 mmol/L to 1.14 mmol/L, a reduction of only 0.12 mmol/L. After perfusion, the concentration of pathologic LDL was reduced to the normal range (≤4.11 mmol/L) in patients with HLP. Although the concentration of HDL was significantly decreased, it still remained in the normal range (>1.15 mmol/L for women and >0.9 mmol/L for men). The reduction of HDL was only about one-seventh that of LDL, indicating a satisfactory therapeutic effect of Fe_3_O_4_@SiO_2_@Azo-COOH for HLP.

### Studies on binding relationship

To assess the binding relationship of Fe_3_O_4_@SiO_2_@Azo-COOH during adsorption, the interaction between the adsorbent and lipoproteins in serum was investigated. The adsorption rate of the adsorbent was determined by fitting the adsorption kinetic equation. The adsorption data of Fe_3_O_4_@SiO_2_@Azo-COOH in HLP patient’s serum was nonlinearly fitted with the pseudo-first-order (2) and pseudo-second-order (3) kinetic equations, which could be expressed as follows:

(2) $$Q_{t}=Q_{e}(1-e^{-k_{1}t})\;$$

(3) $$Q_{t}=k_{2}Q_{e}^{2}t/\left(1+k_{2}Q_{e}t\right)$$

where *Q_e_* and *Q_t_* represent the binding efficiency (mg/g) of the adsorbent at equilibrium and time *t*, respectively; *k*_1_ and *k*_2_ are the equilibrium rate constants for the pseudo-first-order and pseudo-second-order models, respectively.

In the pseudo-first-order adsorption model, the adsorbent binds to the adsorbate through physical interaction with a one-to-one single layer of attachment. Conversely, in the pseudo-second-order adsorption model, the binding interaction leans more towards chemical forces involving electron pair sharing or electron transfer between the adsorbent and the adsorbate. Figure 3B showed the nonlinear fits of the experimental values in serum, with the associated kinetic parameters acquired from the equations presented in Supplementary Table S3. For Fe_3_O_4_@SiO_2_@Azo-COOH, possessing both hydrophobicity and electronegativity, the combination between the nanoadsorbent and the pathologic lipids exhibited a rapid rate during the first 30 min, followed by a gradual decrease over the subsequent 150 min, reaching a maximum adsorption efficiency at 180 min. Both kinetic models characterized the adsorption process well, as indicated by the correlation coefficient (*R*^2^) and the comparison between the experimentally measured values and the *Q_e_* derived from the kinetic equations. This suggests that the binding of Fe_3_O_4_@SiO_2_@Azo-COOH with lipids involved both physisorption and chemisorption. Specifically, in the case of LDL, both adsorption model equations yielded similar *R*^2^ values, and the lipid bounding amounts of both kinetic models were close to the experimental measurements, indicating the presence of both chemical and physical interaction during the clearance process of Fe_3_O_4_@SiO_2_@Azo-COOH. This confirmed that the prepared nanoadsorbent exibited both hydrophobic and electrostatic forces during adsorption, owing to the co-existence of azobenzene functional monomers and carboxyl groups on Fe_3_O_4_@SiO_2_@Azo-COOH.

The thermodynamic properties of the adsorption process were evaluated by nonlinear fitting of adsorption isotherms. The adsorption results were fitted by the Langmuir isotherm model (4) and Freundlich isotherm model (5) using the following equations:

(4) $$Q_{e}=Q_{m}C_{e}/(K_{L}+C_{e})$$

(5) $$Q_{e}=K_{F}C_{e}^{1/n}$$

where *C_e_* represents the equilibrium level (mmol/l) of the adsorbate; *Q_e_* indicates the equilibrium binding quantity(mg/g); *Q_m_* is the maximum binding efficiency (mg/g); *K_L_*, *K_F_* and 1/*n* are the constants.

The nonlinear fitting data were shown in Figure 3C–F. The associated kinetic parameters obtained from the above equations were presented in Supplementary Table S4. Both the Langmuir isotherm model and Freundlich isotherm model accurately described the binding process of Fe_3_O_4_@SiO_2_@Azo-COOH with blood lipids, suggesting both monolayer and multilayer adsorption during the adsorption process. Physisorption processes, due to nonspecific physical interactions, are characterized by multilayer adsorption. Conversely, chemisorption processes, attributed to specific chemoselectivity, are characterized by monolayer adsorption. According to the results of linear fitting, both isotherm models effectively explained the adsorption of LDL, TCH and TG, suggesting the existence of dual chemical and physical effects in the adsorption process. Moreover, only the Freundlich isotherm model could be applied to characterize the adsorption process of HDL. The $R_{L}^{2}$ value of HDL was relatively low compared to LDL, TCH and TG, which might be directly related to the predominance of physical adsorption. According to these resultes, the isothermal adsorption curve fitting outcomes were consistent with our presumed adsorption mechanism, reaffirming the feasibility of Fe_3_O_4_@SiO_2_@Azo-COOH as an ideal adsorbent for HLP.

### Photocontrolled adsorption

The azobenzene group undergoes *cis-trans* isomerization under different light irradiations, corresponding to different dipole moments (5.5 Å for the *cis*-isomer, 9.0 Å for the *trans*-isomer). This difference in dipole moments leads to changes in hydrophilicity and hydrophobicity of the adsorbent surface. We confirmed the existence of the above phenomenon through changes in UV–Vis absorption (Figure 2) and surface WCA (Figure 1E) of Fe_3_O_4_@SiO_2_@Azo-COOH (19.33% reduction) under different illumination conditions. The adsorption properties of photoresponsive adsorbent were further demonstrated by their photoregulated binding and release of blood lipids. To investigate the light-controlled mechanism of Fe_3_O_4_@SiO_2_@Azo-COOH more precisely, the photo-switched adsorption–desorption of adsorbent in a pure LDL adsorbate solution was first investigated.

Figure 5A illustrated the transformation of the binding ability of Fe_3_O_4_@SiO_2_@Azo-COOH with LDL under successive photoconversion conditions. Light-controlled cyclic adsorption experiments were conducted combining adsorption saturation time and the photoresponse time of the adsorbent, which showed reversible binding of the adsorbent containing azobenzene chromophores during photoconversion. Typically, the Fe_3_O_4_@SiO_2_@Azo-COOH nanoadsorbent and LDL solution were mixed with an adsorption ratio of 10:1 (mg: ml) and incubated in the dark for 2 h at 37°C. The results showed that the Fe_3_O_4_@SiO_2_@Azo-COOH nanoadsorbent exhibited high adsorption performance to LDL, with a binding efficiency of 15.06 mg/g. Then, after transforming the light source, the mixture was irradiation under UV light for 2 h at 37°C, resulting in a significant release of the adsorbate from Fe_3_O_4_@SiO_2_@Azo-COOH into the adsorption solution. Following 2 h of UV light incubation, the adsorption capacity of LDL reduced to 5.64 mg/g. The subsequent reverse thermal isomerization of the nanoadsorbent without UV light resulted in an improvement in the binding efficiency of 14.59 mg/g, which was essentially recovered to the original level. This indicated that the photoisomerization behaviors of azobenzene directly affected the hydrophilicity of Fe_3_O_4_@SiO_2_@Azo-COOH, leading to changes in the adsorption capacity without inducing other side reactions. This procedure was repeated 5 times, demonstrating the reversibility of binding caused by azobenzene-chromophore photoswitching during the successive adsorption–desorption cycle, where LDL underwent cyclic binding and release. This above phenomenon was further verified by real-time monitoring of the photocontrolled adsorption process through TEM and FTIR. The TEM images (Figure 5B and Supplementary Figure S8) indicated that during the cycling process, the adsorbent in both states (azobenzene in *trans* and *cis* structures) adsorbed LDL. However, judging from the amount of lipid proteins attached to the surface, the quantity of LDL adhered to the adsorbent of the *cis* surface was much less than that of the *trans* surface, which was the most visible indication of photocontrolled binding and release. The FTIR spectra (Figure 5C) showed that LDL was only attached to the surface of the adsorbent with no influence of the LDL original characteristics, only differing in the amount of attachment between the two states. In Figure 5C, the amide I peak at 1665 cm^−1^ blueshifted to 1637 cm^−1^, confirming that the secondary structure of LDL had transformed from α-helical to predominantly β-sheet structure. In addition, the peak at 3338 cm^−1^ belonging to the N-H of amide I shifted downward to 3273 cm^−1^. The increment and blue-shift of amide II peak at 1542 cm^−1^ also suggested that the formation the hydrogen bonds (N-H···X (O, N)) could influence the movement of the H-donor group peaks to lower wavelengths [43].

To further demonstrate the clinical application value of the adsorbent, the adsorbent with light-controlled adsorption–desorption performance was applied for the removal of lipids from HLP serum (Figure 5D). It was found that the adsorption of LDL exhibited a photocontrolled-change rate of 72.2%, indicating a significant reduction in LDL levels through light-controlled adsorption. Similarly, the adsorption of TG and TCH also displayed good cyclic adsorption performance, with photocontrolled change rates of 49.08% and 48.36%, respectively. Although the adsorption of HDL also showed cyclic performance, its adsorbent capacity was extremely low, further demonstrating the nanoadsorbent's excellent selectivity-adsorption properties. The adsorbent Fe_3_O_4_@SiO_2_@Azo-COOH enabled “intelligent” photocontrolled adsorption–desorption of blood lipids in HLP serum. Furthermore, the magnetic responsiveness of the adsorbent allowed rapid enrichment and separation of the adsorbent, significantly reducing the cost of adsorbent use. This biomimetic nanoadsorbent, thus, emerges as a potential option for patients with severe HLP, offering promising prospects for effective lipid removal in clinical settings.

### Recycling and reusability of the adsorbent

**Figure 6.**
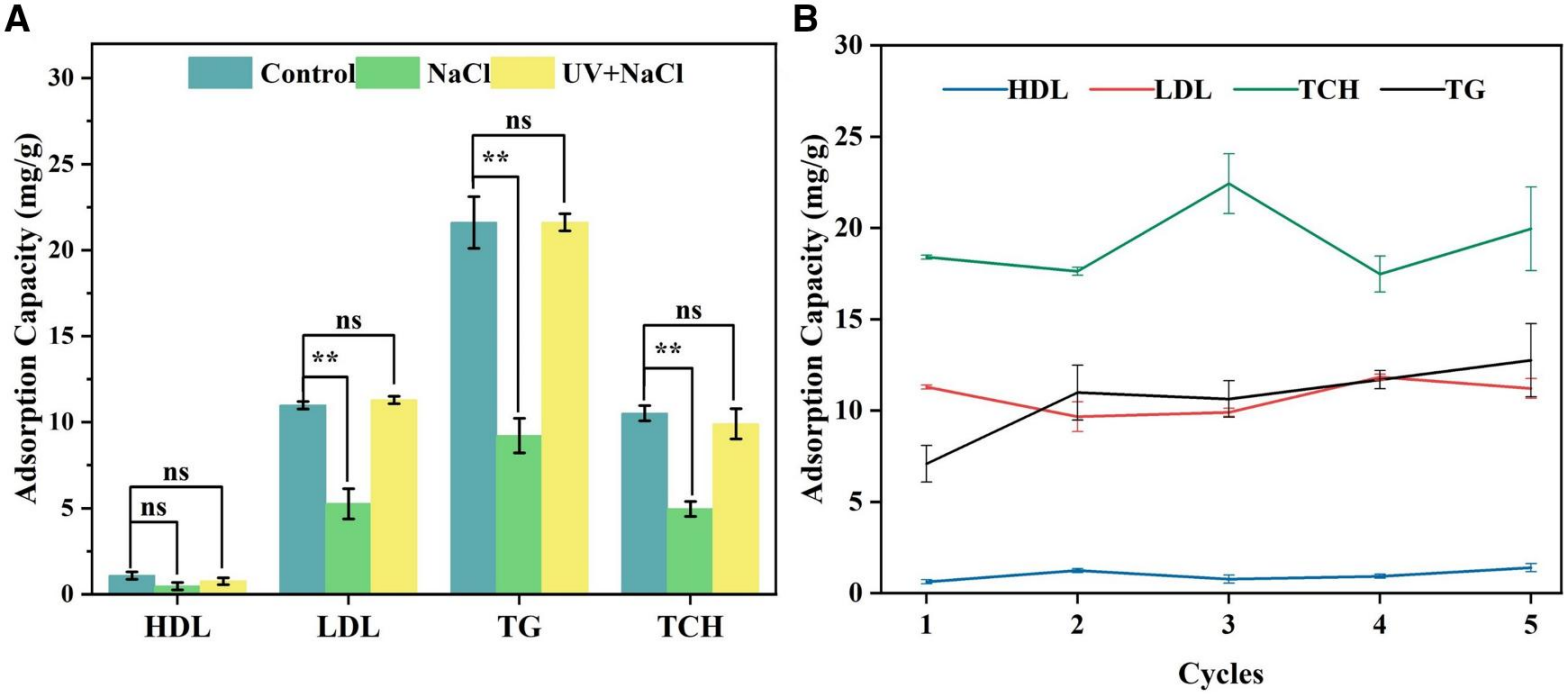
Recovery and reuse of the prepared nanoadsorbent. (**A**) Effect of different recovery methods on the adsorption capacity of Fe_3_O_4_@SiO_2_@Azo-COOH in HLP serum. (*n* = 3, **P < 0.01); (**B**) cyclic adsorption capacity of blood lipids on Fe_3_O_4_@SiO_2_@Azo-COOH in HLP serum (*n* = 3).

Drawing upon the synthesis concept of Fe_3_O_4_@SiO_2_@Azo-COOH and our understanding of the adsorption relationship, we implemented a recycling strategy for the nanoadsorbent, fully capitalizing on its biomimetic properties. The magnetic responsiveness of the nanoadsorbent was first utilized for particle enrichment and separation, followed by complete regeneration of the adsorbent using a combination of the adsorbent's photoresponsive properties and an antistatic solvent. Our results demonstrated that the recovery and regeneration of adsorbent were fully realized through magnetic responsiveness and influence of light, as well as the use of an antistatic solvent. This method proved to be highly reproducible (Figure 6A), maintaining a recovery rate of 99.4% even after five cycles of reuse (Figure 6B). During the regeneration process, we capitalized on the magnetic responsiveness of the nanoadsorbent to achieve rapid separation. Additionally, we employed the intrinsic photoresponsive characteristic of Fe_3_O_4_@SiO_2_@Azo-COOH to disrupt the hydrophobic interactions between the blood lipids and the nanoadsorbent. Furthermore, an antistatic solvent (NaCl) was used to neutralize the electrostatic effects between the blood lipids and the nanoadsorbent [37, 44]. This comprehensive approach ultimately led to complete green regeneration, ensuring the adsorbent could be reused effectively and sustainably. Although it was established in our experiment that both photoresponsiveness and antistatic action alone could not achieve full regeneration of the adsorbent, our environmentally friendly and controllable procedure demonstrated promising application prospects by combining these approaches, resulting in full regeneration without secondary pollution and through a simple process.

### Biocompatibility

The biocompatibility of the nanoadsorbent was systematically evaluated through blood routine analysis, anticoagulant activity analysis, hemolysis test and cytotoxicity test, with Fe_3_O_4_@SiO_2_ nanoparticles serving as a control. As depicted in Supplementary Figure S9, the prepared adsorbent exhibited almost negligible cytotoxicity (cytotoxicity grade was 1), displayed negligible adverse effects on blood cells, and induced no hemolysis or clotting, suggesting satisfactory biocompatibility and clinical safety.

## Conclusion

We have successfully developed a bionic nanoadsorbent Fe_3_O_4_@SiO_2_@Azo-COOH, capable of selectively and intelligently removing LDL, TG and TCH from HLP serum while sparing HDL. This nanoadsorbent exhibited remarkable binding efficiency, with values of 10.93, 21.26 and 9.80 mg/g for LDL, TG and TCH, respectively. Moreover, its adsorption capacity for HDL was significantly lower at only ∼0.77 mg/g, underscoring its selective binding performance for LDL and resistance to atheroprotective HDL. Importantly, the adsorption performance of Fe_3_O_4_@SiO_2_@Azo-COOH surpassed that of previously reported adsorbents [15, 22, 45, 46]. Additionally, Fe_3_O_4_@SiO_2_@Azo-COOH displayed excellent adsorption–desorption behaviour for LDL, TG and TCH due to its photocontrolled irradiation capabilities, thereby reducing synthesis costs and resource wastage. When combined with micromagnetic microfluidics, dynamic perfusion experiments showed a significant reduction in serum LDL content, suggesting its potential application in blood perfusion therapies. The dual photomagnetic responsiveness of the nanoadsorbent facilitates its use in hemoperfusion for clearing pathogenic blood lipids from HLP patients' serum. Considering the easy availability of azobenzene functional monomers through molecular design synthesis and the efficient synthesis of bionic adsorbent, we have developed a modular renewable nanoadsorbent for hemoperfusion. This innovation holds promise for easily and efficiently clearing harmful substances from blood. However, the limitations inherent to nanoparticles currently hinder the direct application of the adsorbent in clinical treatment. In the future, our focus will be on optimizing performance through the construction of adsorbent carriers to enable clinical blood perfusion.

## Supplementary Material

rbae045_Supplementary_Data
