# Supplementary material for: Dual stimulus-responsive renewable nanoadsorbent for selective adsorption of low-density lipoprotein in serum
Source: Regen Biomater. 2024 Apr 29;11:rbae045. doi: 10.1093/rb/rbae045 (PMC11153342; doi:10.1093/rb/rbae045)
Supplement: rbae045_Supplementary_Data [file rbae045_supplementary_data.docx]

*Supplementary Information*

**Dual stimulus-responsive renewable nanoadsorbent for selective adsorption of LDL in serum**

Chen Guo, Xinbang Jiang,Xiaofang Guo, Zhuang Liu, Biao Wang, Yunzheng Du, Ziying Tian, Zimeng Wang, Lailiang Ou*

Key Laboratory of Bioactive Materials, Ministry of Education, College of Life Science, Nankai University, Tianjin 300071, China.

**Experimental section**

**Reagents and materials**

Tetrahydrofuran (THF, Aladdin, Shanghai, China, AR) was dried with anhydrous sodium sulfate and then distilled under vacuum. Methanol (Tianjin Jiangtian Chemical, Tianjin, China,, AR) was distilled prior to use. 2,5-dioxopyrrolidin-1-yl (*E*)-4-((4-((10-(acryloyloxy)decyl)oxy)phenyl)diazenyl)benzoate (AL_10_-OSU) were synthesized according to the methods in the literature[1]. Triethylamine (TEA, Tianjin Jiangtian Chemicals, Tianjin, China, 99%), tetraethyl orthosilicate (TEOS, Aladdin Shanghai, China,, 98%), (3-aminopropyl)triethoxysilane (APTES, Sigma St. Loius, MO, USA, 98%), 3-mercaptopropionic acid (Aladdin, Shanghai, China, 98%).

All human serum samples were provided from HLP patients in Second Hospital Affiliated to Tianjin Medical University and were collected following experimental protocols reviewed and approved by the Ethics Committee of Nankai University (Tianjin, China) (ethics number NKUIRB2022091). The work described here has been carried out in accordance with The Code of Ethics of the World Medical Association, and an informed consent was obtained for experimentation with all donors.

**Synthesis of magnetic nanoparticles (MNPs)**

FeCl_3_·6H_2_O (0.43 g) and sodium acetate (1.2 g) were dissolved in ethylene glycol (14 mL) and the solution was stirred for 30 min at room temperature until uniformly dispersed. The mixture solution was then transferred into an autoclave, and the reaction was performed at 200°C for 8 h. After the reaction, the product was separated magnetically, washed with ethanol and distilled water, followed by drying at 60^o^C under vacuum for 48 h to obtain a brownish-black powder[2].

**Preparation of silica-coated core-shell structure MNPs (Fe_3_O_4_@SiO_2_)**

The silica coated magnetic core-shell nanoparticles (Fe_3_O_4_@SiO_2_) were synthesized via the Stöber method[3]. Typically, MNPs (0.3000 g) were dispersed in the ethanol-water solution (300 mL) via ultrasonication. Then ammonia solution (6 mL) and TEOS (0.642 mL) were added consecutively to the reaction mixture, and the reaction was performed at 25°C for 6 h. The resulting particles were collected and washed successively with ethanol, deionized water, followed by vacuum drying at 60°C for 24 h.

**Preparation of one-pot amino-modified silica-coated core-shell structure MNPs (Fe_3_O_4_@SiO_2_-NH_2_)**

The amino-modified silica-coated magnetic core-shell nanoparticles (Fe_3_O_4_@SiO_2_) were synthesized via a modified Stöber method[4-6]. Firstly, the MNPs (0.3 g) were dispersed in deionized water (100 mL), with ethanol (240 mL) and ammonia (240 μL) added and stirred thoroughly to ensure even dispersion. Subsequently, TEOS (2400 μL) was added dropwise with sufficient stirring for 30 min, followed by the addition of APTES (2400 μL) at room temperature for 12 h. The reaction was carried out by magnetic separation of the MNPs. After the reaction, the products with core-shell structure were obtained by magnetic separation with methanol, deionized water and ethanol until clear and transparent, showing a weight increase of 14.4% compared to Fe_3_O_4_.

**SEM characterization**

The morphologies, particle sizes, size distributions and surface structures of the samples were characterized with a scanning electron microscope (SEM, MERLIN Compact, ZEISS, Germany) (**Fig.** **S2** and **Table** **S1**). All of the SEM size data reflect the averages of more than 100 particles, which were calculated by using the following formulas according to previously reported method[7] :

*D*_n_=$\sum_{i=1}^{k} n_{i}D_{i}$**/**$\sum_{i=1}^{k} n_{i} \text{ (S1)}$

*D_w_*=$\sum_{\text{i}\text{=1}}^{\text{k}} \text{n}_{\text{i}}\text{D}_{\text{i}}^{\text{4}}$**/**$\sum_{\text{i}\text{=1}}^{\text{k}} \text{n}_{\text{i}}\text{D}_{\text{i}}^{\text{3}} \text{(S2)}$

*U*=$D_{w}$**/**$\text{D}_{\text{n}} \text{(S3}$)

where *D*_n_ was the number-average diameter. *D_w_* was the weight-average diameter. U was the size distribution index. *k* was the total number of the measured particles. *D_i_* was the particle diameter of the *i*th polymer particle, and n*_i_* the number of the particles with a diameter *Di*.

# Biocompatibility assessment of adsorbents[8]

For routine blood test, fresh blood from healthy donor was collected with Ethylene Diamine Tetraacetic Acid (EDTA) test tube. 0.6 mL of freshly drawn blood was mixed with the prepared adsorbent and incubated at 37℃. After 1 h incubation, the blood cells were separated, and the white blood cells (WBC), red blood cells (RBC), hematocrit (HCT), platelets (PLT) and hemoglobin (HGB) were examined (Sysmex XE-2100, Kobe, Japan).

For anticoagulant activity, fresh blood of healthy donor was collected with sodium citrate anticoagulated blood collection tubes. The blood samples were centrifuged at 3000 rpm/min for 5 min to obtain platelet-poor plasma (PPP). 0.5 mL of PPP was added to 0.0050 g of adsorbents and incubated at 37°C with gently oscillation. Pure PPP without samples was used as controls. The anticoagulant properties of Fe_3_O_4_@SiO_2_ and Fe_3_O_4_@SiO_2_@Azo-COOH were elvaluated by activared partial thromboplastin time (APTT), prothrombin time (PT), thrombin time (TT) and fibrinogen (FIB). The measurements were performed by a semiautomatic blood coagulation analyzer CA-50 (Sysmex Corporation, Kobe, Japan).

For hemolysis analysis, fresh blood of healthy rabbits was anticoagulated by adding 2% potassium oxalate and diluted with 0.9% NaCl solution in order to produce the RBC suspension (Synergy 4, BioTek, America). The RBC suspensions (0.1 mL for each sample) were severally mixed with: (a) 5 mL of 0.9% NaCl solution as a negative control; (b) 5 mL of deionized water as positive control; (b) 5 mL of 0.9% NaCl solution and 10 mg of Fe_3_O_4_@SiO_2_ as control group; (d) 5 mL of 0.9% NaCl solution and 10 mg of Fe_3_O_4_@SiO_2_@Azo-COOH as experimental group. All the tubes are incubated at 37°C for 60 min, blood cells were removed by centrifugation (3000 rpm/min) and the supernatants were evaluated at 545 nm for the release of hemoglobin. The extent of hemolysis was represented as hemolysis ratio (%) which is calculated by equation (S4):

$\text{Hemolysis ratio }\left( \text{\%} \right)\text{=}\frac{\text{OD}_{\text{Sample}}\text{-}\text{OD}_{\text{Negative Control}}}{\text{OD}_{\text{Positive Control}}\text{-}\text{OD}_{\text{Negative Control}}}\text{×100}$ (S4)

where $\text{OD}_{\text{Sample}}$ was the supernatant from the blood incubated with Fe_3_O_4_@SiO_2_ or Fe_3_O_4_@SiO_2_@Azo-COOH. $\text{OD}_{\text{Positive Control}}$ was the supernatant from the blood incubated with deionized water. $\text{OD}_{\text{Negative Control}}$ was the supernatant from the blood incubated with normal saline.

For cytotoxicity test, standard Cell Counting Kit-8 (CCK-8) assay was employed to evaluate cell viability. The conditioned media was prepared by incubation 200 mg of the Fe_3_O_4_@SiO_2_ or Fe_3_O_4_@SiO_2_@Azo-COOH (as control) into 20 mL of cell culture media containing 10% fetal calf serum, 90% of DMEM and 1% of antibiotics-antimycotics at 37°C for 48 h. The cell line used in cytotoxicity test was human umbilical vein endothelial cells (HUVECs) with a seeding density at about 30,000/mL. HUVECs was seeded into a 96 well plate at seeding volume of 100 μL and incubated in cell culture media in the incubator at 37 °C and 5% CO_2_ for 24 h. The cell culture media was then replaced with conditioned media to evaluate the cytotoxicity of the prepared adsorbent. After culture for 24 h, 10 μL of CCK-8 solution was added into each well of the plate, and then the cells were cultured in the dark for 1 h. The absorbance of the solution was measured using a multifunctional enzyme marker at a wavelength of 450 nm. The cell viability was calculated by equation (S5):

$\text{Cell Viability = }\frac{\text{X}}{\text{X}_{\text{0}}}\text{×100\%}$ (S5)

where $\text{X}$ was the $\text{OD}$ value of cells cultured in conditioned media (Fe_3_O_4_@SiO_2_ or Fe_3_O_4_@SiO_2_@Azo-COOH). $\text{X}_{\text{0}}$ was the $\text{OD}$ value of cell incubated in cell culture media.

**Statistical analysis.**

All experiments were conducted in triplicate. Values was presented as mean±standard deviation (n=3) in associated tables and figures. Statistical significance was displayed as * when 0.01<P<0.05, ** when 0.001<P<0.01, *** when P<0.001, **** when P<0.0001 ns when P>0.05 by hypothesis T-test.

**Reference**

1. Guo C, Gao J, Ma S, Zhang H. Efficient preparation of chemically crosslinked recyclable photodeformable azobenzene polymer fibers with high processability and reconstruction ability via a facile post-crosslinking method. *European Polymer Journal* 2020;139:109998.

2. Jiang X, Zhang X, Guo C, Yu Y, Ma B, Liu Z, Chai Y, Wang L, Du Y, Wang B, Li N, Dong D, Li Y, Huang X, Ou L. Protein corona-coated immunomagnetic nanoparticles with enhanced isolation of circulating tumor cells. *Nanoscale* 2022;14:8474-8483.

3. Yu Y, Ma B, Jiang X, Guo C, Liu Z, Li N, Chai Y, Wang L, Du Y, Wang B, Li W, Ou L. Amphiphilic shell nanomagnetic adsorbents for selective and highly efficient capture of low-density lipoprotein from hyperlipidaemia serum. *J Mater Chem B* 2022;10:4856-4866.

4. Xu S, Zou Y, Zhang H. Well-defined hydrophilic "turn-on"-type ratiometric fluorescent molecularly imprinted polymer microspheres for direct and highly selective herbicide optosensing in the undiluted pure milks. *Talanta* 2020;211:120711.

5. Zhang JM, Zhai SR, Zhai B, An QD, Tian G. Crucial factors affecting the physicochemical properties of sol–gel produced Fe3O4@SiO2–NH2 core–shell nanomaterials. *Journal of Sol-Gel Science and Technology* 2012;64:347-357.

6. Zhang J, Zhai S, Li S, Xiao Z, Song Y, An Q, Tian G. Pb(II) removal of Fe3O4@SiO2–NH2 core–shell nanomaterials prepared via a controllable sol–gel proce. *Chemical Engineering Journal* 2013;215-216:461-471.

7. Lu X, Zheng C, Zhang H. Improvement of surface hydrophilicity and biological sample-compatibility of molecularly imprinted polymer microspheres by facile surface modification with α-cyclodextrin. *European Polymer Journal* 2019;115:12-21.

8. Guo C, Yu Y, Jiang X, Ma B, Liu Z, Chai Y, Wang L, Wang B, Du Y, Li N, Fan H, Ou L. Photorenewable Azobenzene Polymer Brush-Modified Nanoadsorbent for Selective Adsorption of LDL in Serum. *ACS Appl Mater Interfaces* 2022;14:34388-34399.

# ^1^H NMR characterization


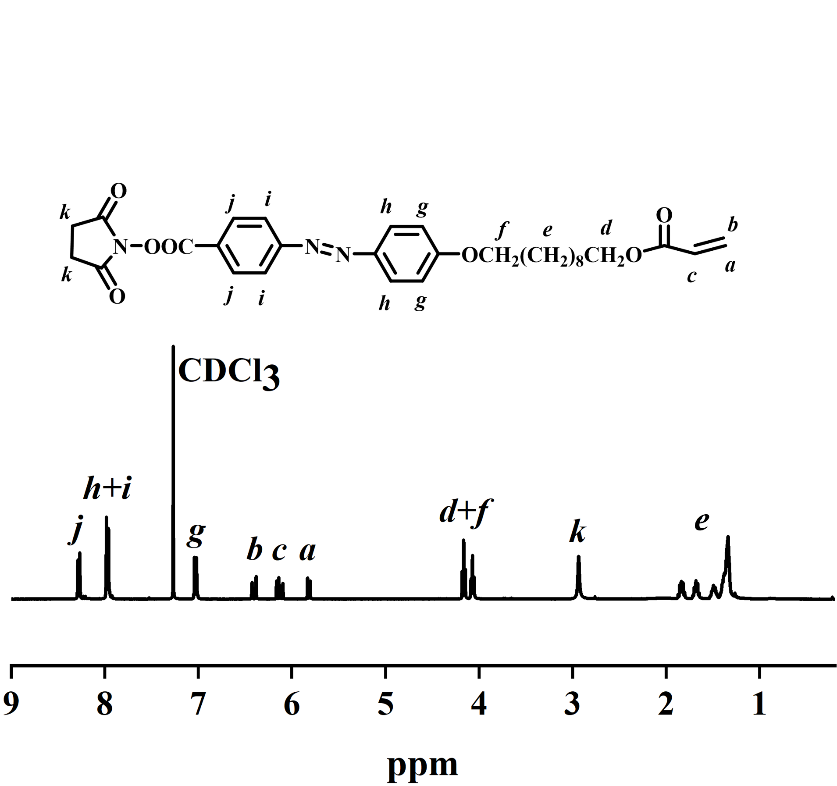


**Fig. S1** ^1^H NMR spectra of AL_10_-OSU in CDCl_3_, the chemical shifts and peak integartions of all the protons in the monomer are in excellent agreement with its expected structure.

# SEM characterization


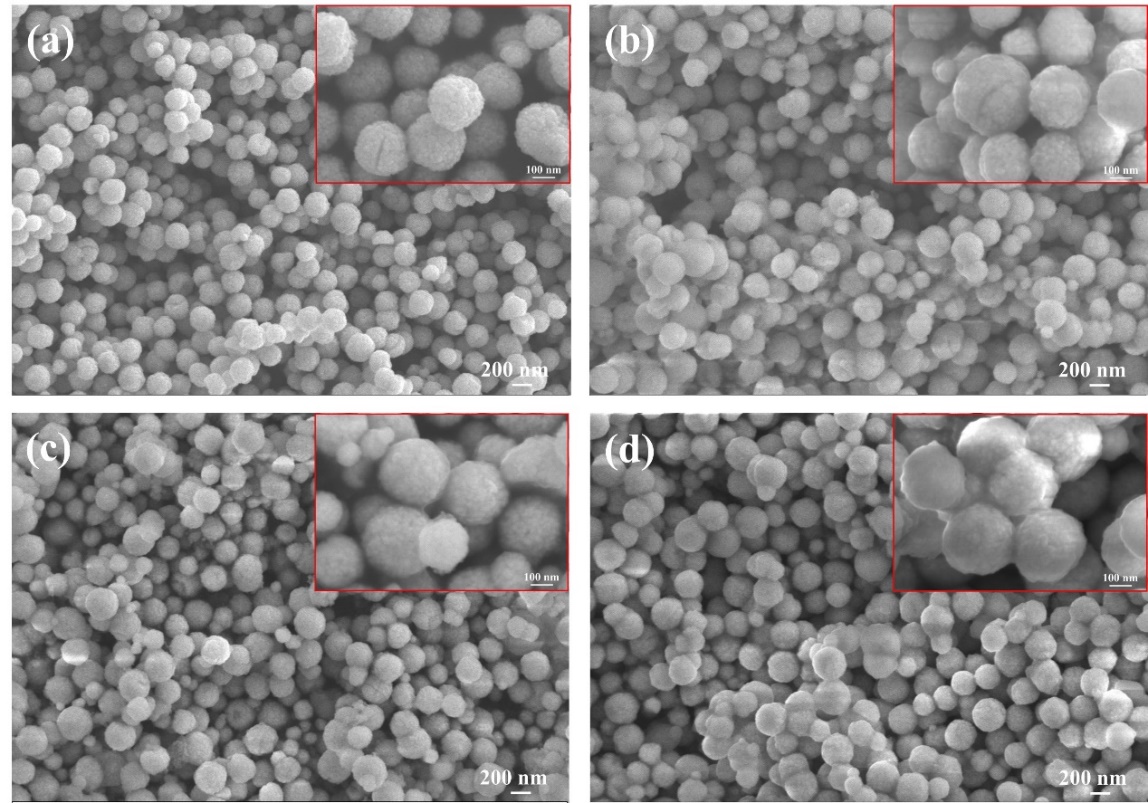


**Fig. S2** SEM images of (a) Fe_3_O_4_, (b) Fe_3_O_4_@SiO_2_-NH_2_, (c) Fe_3_O_4_@SiO_2_@Azo, and (d) Fe_3_O_4_@SiO_2_@Azo-COOH.

# TEM characterization


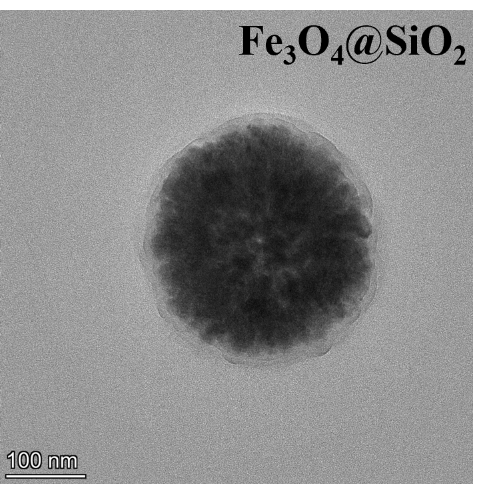


**Fig. S3** TEM image of Fe_3_O_4_@SiO_2_ (with higher magnification).

**Aqueous dispersion stability test**


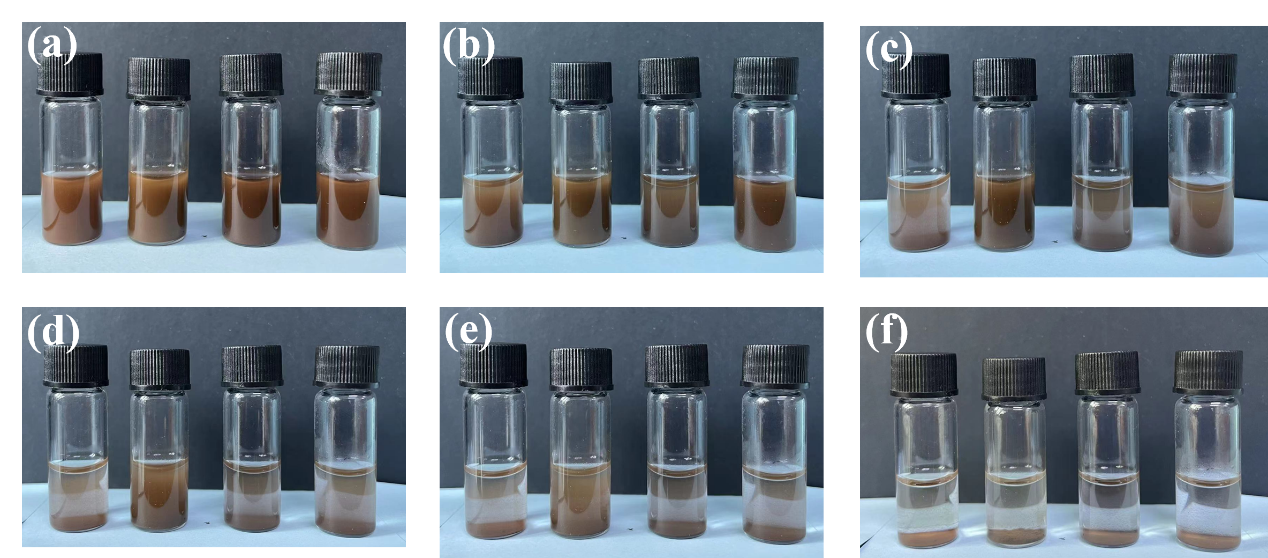


**Fig. S4** Detailed photographs of the ultrasonically dispersed aqueous mixtures (1.0 mg/mL) after their being settled down for (a) 0 min, (b) 15 min, (c) 30 min, (d) 45 min, (e) 60 min, and (f) 135 min, respectively. The samples located from left to right in each photograph are Fe_3_O_4_, Fe_3_O_4_@SiO_2_-NH_2_, Fe_3_O_4_@SiO_2_@Azo, and Fe_3_O_4_@SiO_2_@Azo-COOH.

# TG characterization

**
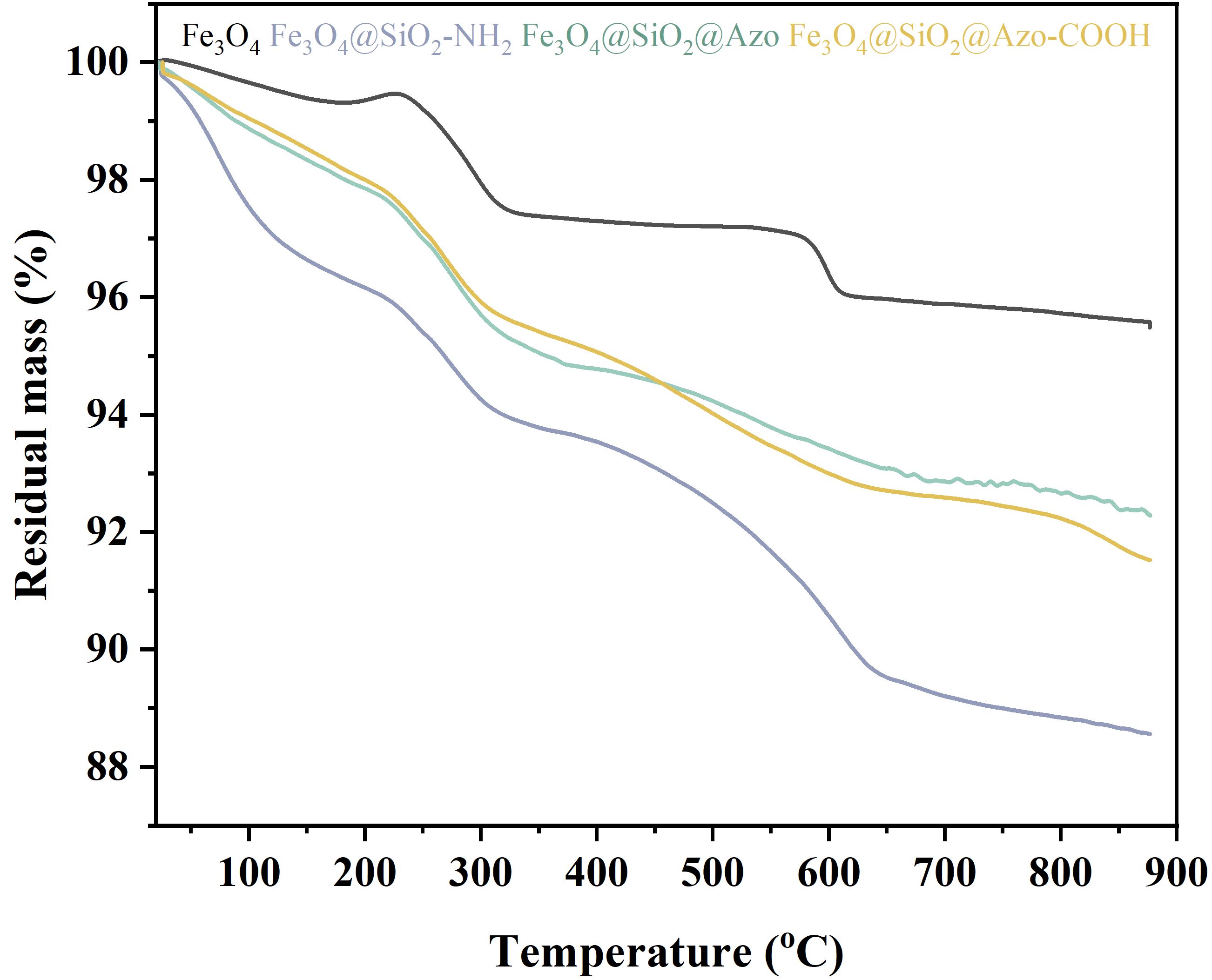
**

**Fig. S5** TG characterization of Fe_3_O_4_, Fe_3_O_4_@SiO_2_-NH_2_, Fe_3_O_4_@SiO_2_@Azo, and Fe_3_O_4_@SiO_2_@Azo-COOH. (The testing was performed with the protection of nitrogen atmosphere at a heating rate of 10°C /min.)

# Superparamagnetic characterization


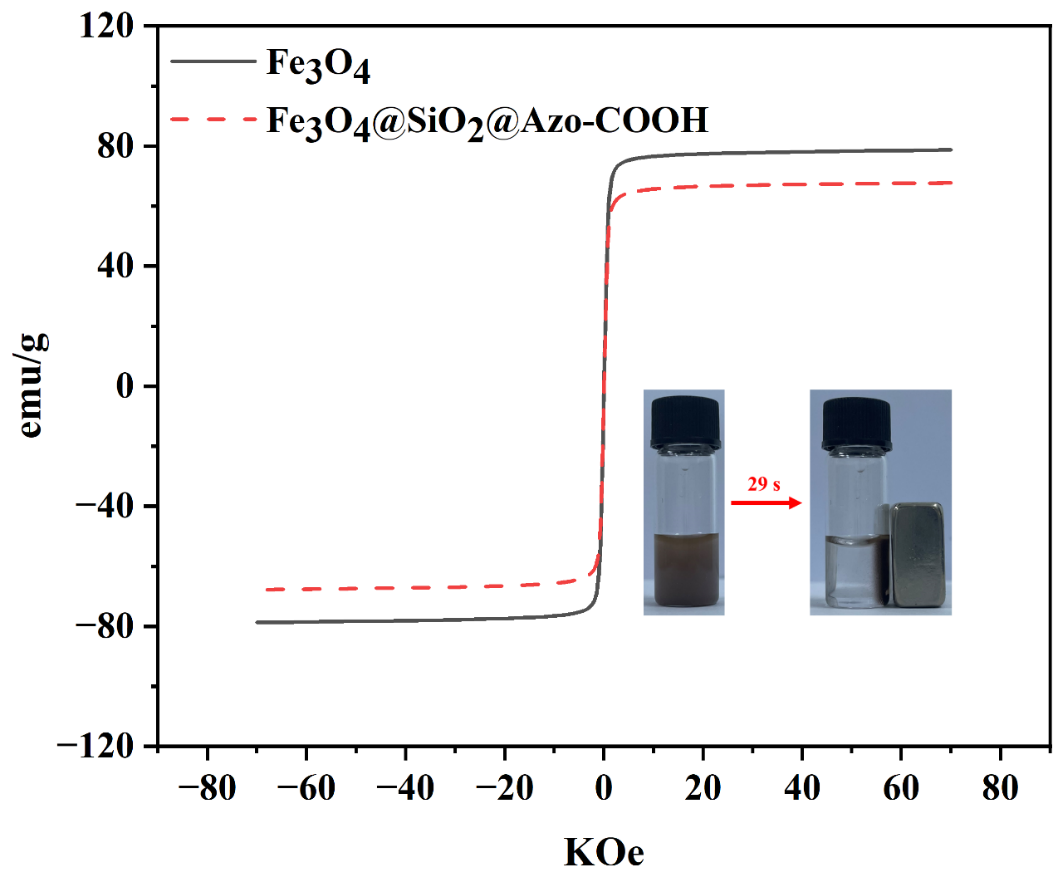


**Fig. S6** Magnetization curves of Fe_3_O_4_ and Fe_3_O_4_@SiO_2_@Azo-COOH.

**Hemoperfusion physical drawing**


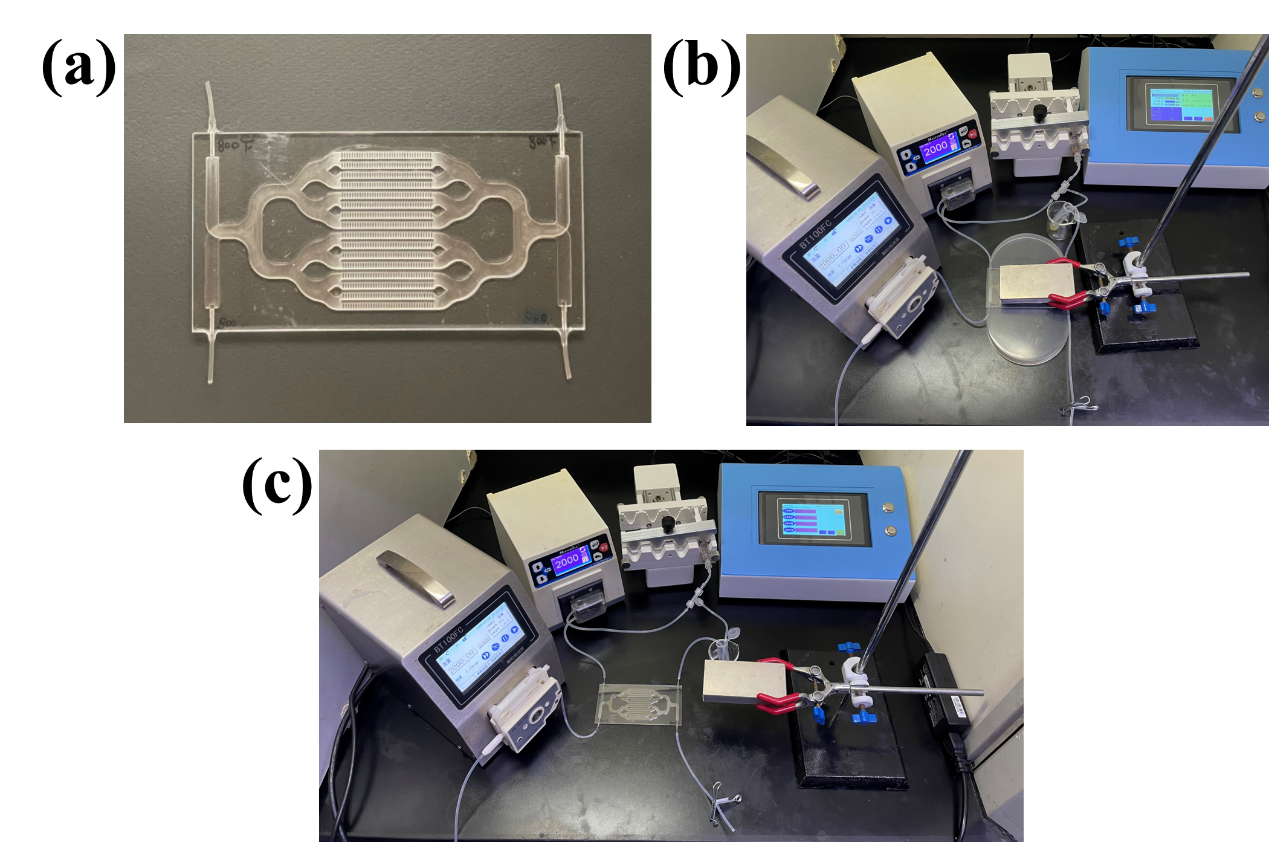


**Fig. S7** (a) Photograph of the microfluidic chip; photographs of dynamic adsorption ((b) before and (c) after adsorption).

**TEM characterization of Light-controlled adsorption**


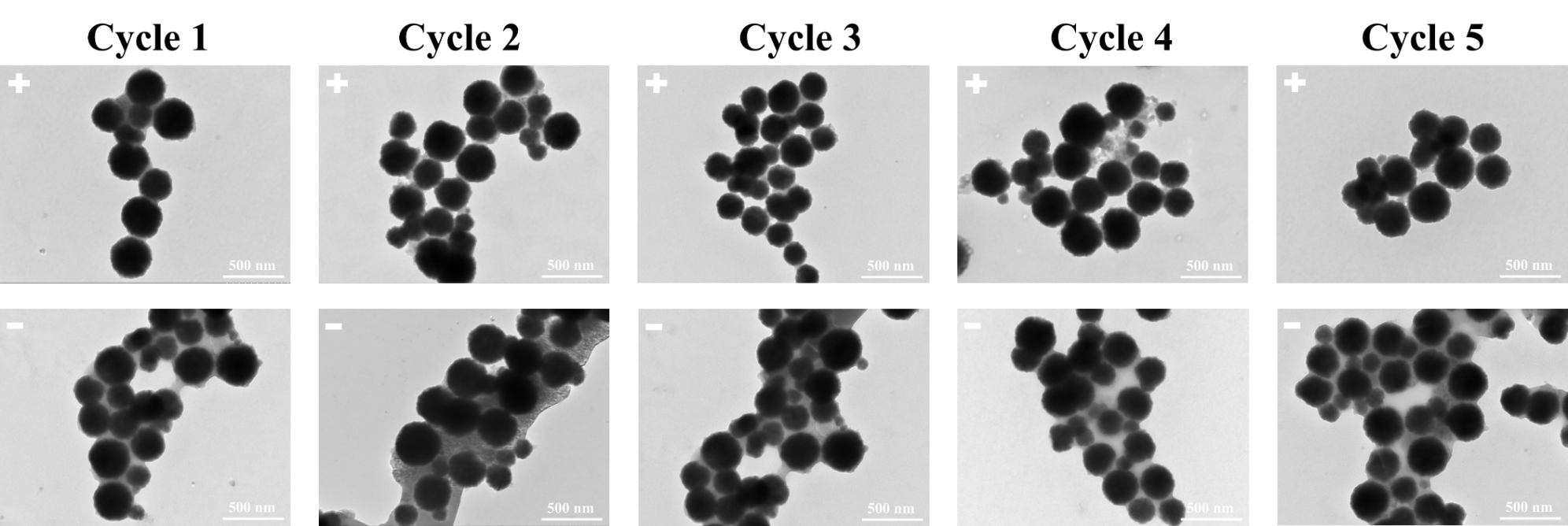


**Fig. S8.** TEM images during light-controlled adsorption (Cycle 3-5) in LDL PBS solution of Fe_3_O_4_@SiO_2_@Azo-COOH.

**Biocompatibility**


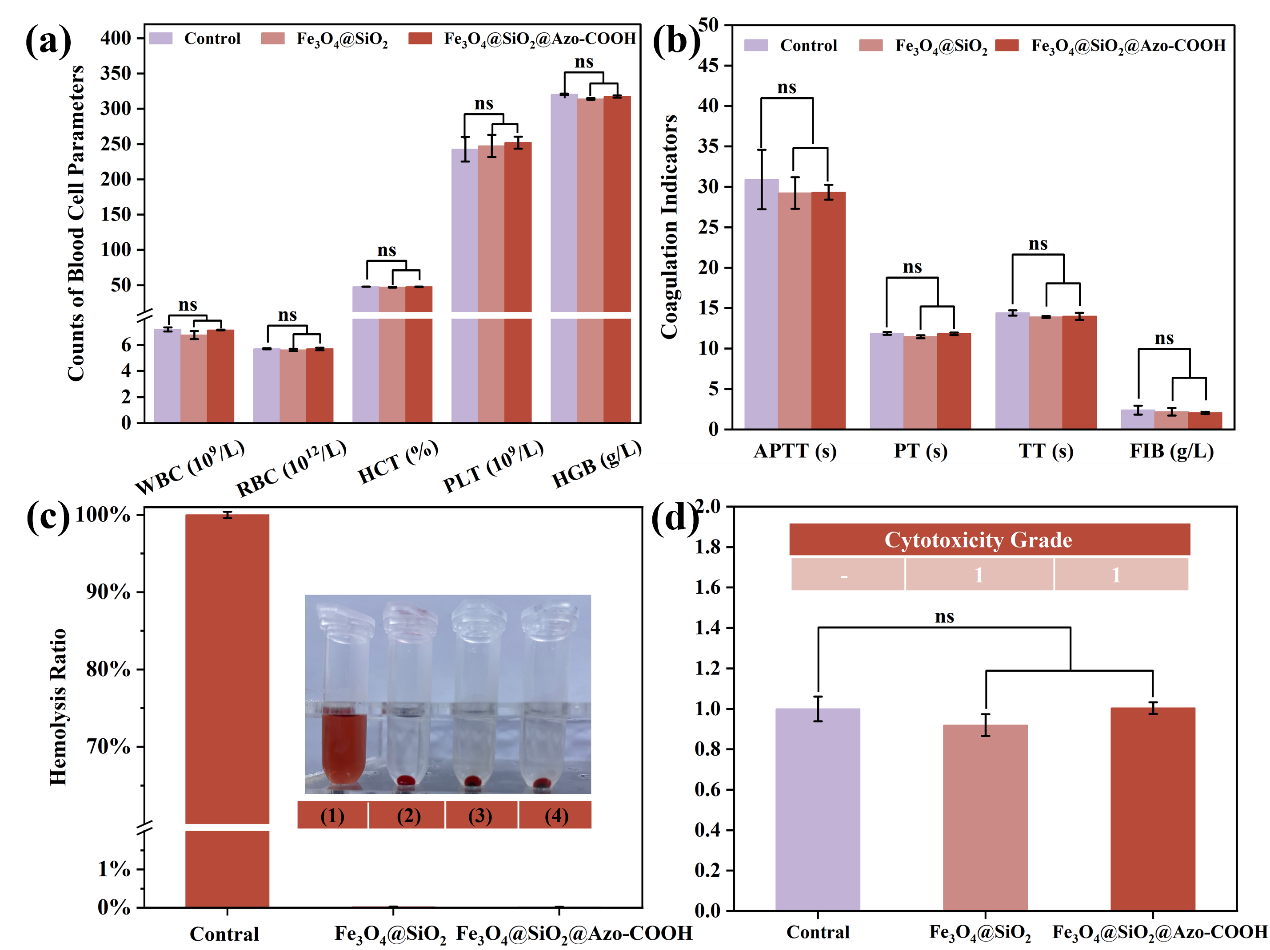


**Fig. S9** Biocompatibility of the prepared nanoadsorbent. (a) blood routine test, (b) coagulation test, (c) hemolysis experiments, and (d) cytotoxicity experiments of Fe_3_O_4_@SiO_2_ and Fe_3_O_4_@SiO_2_@Azo-COOH (n=3).

| **Table S1**  Polymerization data and characterization results of different nanoparticals. | | | | |
| --- | --- | --- | --- | --- |
| Entry | Sample | Weight increase (%)*^a^* | D_n, SEM_ (nm)*^b^* | U*^b^* |
| 1 | Fe_3_O_4_ | - | 225.2 | 1.058 |
| 2 | Fe_3_O_4_@SiO_2_-NH_2_ | 14.4% | 295.5 | 1.124 |
| 3 | Fe_3_O_4_@SiO_2_@Azo | 7.1% | 293.9 | 1.067 |
| 4 | Fe_3_O_4_@SiO_2_@Azo-COOH | 4.9% | 301.3 | 1.057 |

*a* The increased weight percentage of the polymer particles in comparison with their starting polymer particles after each surface modification step.

*b* D_n, SEM_ and U refer to the number-average diameter of the particles and size distribution index of the particles determined by SEM.

| **Table S2**  Nonlinear fitting of experimental data with the relative parameters calculated from Pseudo-first-order and Pseudo-second-order models for Fe_3_O_4_@SiO_2_@Azo-COOH (n=3). | | | | | | | | | | |  |
| --- | --- | --- | --- | --- | --- | --- | --- | --- | --- | --- | --- |
| Blood lipid | Experimental |  | Pseudo-first-order | | |  | | Pseudo-second-order | | |  |
|  | Q_e_ | k_1_ | | R^2^ | Q_e_ | | k_2_ | | R^2^ | Q_e_ | |
| HDL | 0.77 | 0.024 | | 0.972 | 0.818 | | 0.022 | | 0.953 | 0.121 | |
| LDL | 10.93 | 0.008 | | 0.980 | 14.816 | | 0.0002 | | 0.978 | 23.453 | |
| TCH | 21.26 | 0.008 | | 0.952 | 27.01 | | 0.0001 | | 0.946 | 44.18 | |
| TG | 9.80 | 0.015 | | 0.985 | 9.95 | | 0.0009 | | 0.980 | 13.66 | |

| **Table S3**  Nonlinear fitting of experimental data with the relative parameters calculated from Langmuir and Freundlich models for Fe_3_O_4_@SiO_2_@Azo-COOH (n=3). | | | | | | | | |
| --- | --- | --- | --- | --- | --- | --- | --- | --- |
| Blood lipid | Langmuir constant | | |  | | Freundlich constants | | |
|  | Q_m_ | K_L_ | R_L_^2^ | | 1/n | | K_F_ | R_F_^2^ |
| HDL | 0.7734 | - | - | | 0.504 | | 1.121 | 0.894 |
| LDL | 10.93072 | 5.017 | 0.986 | | 1.587 | | 4.408 | 0.976 |
| TCH | 21.2568 | 9.008 | 0.958 | | 1.471 | | 6.392 | 0.941 |
| TG | 9.7964 | 0.911 | 0.984 | | 2.174 | | 9.506 | 0.992 |
